# Genetic Evaluation of the Patients with Clinically Diagnosed Inborn Errors of Immunity by Whole Exome Sequencing: Results from a Specialized Research Center for Immunodeficiency in Türkiye

**DOI:** 10.1007/s10875-024-01759-w

**Published:** 2024-07-02

**Authors:** Baran Erman, Umran Aba, Canberk Ipsir, Damla Pehlivan, Caner Aytekin, Gökhan Cildir, Begum Cicek, Ceren Bozkurt, Sidem Tekeoglu, Melisa Kaya, Cigdem Aydogmus, Funda Cipe, Gulsan Sucak, Sevgi Bilgic Eltan, Ahmet Ozen, Safa Barıs, Elif Karakoc-Aydiner, Ayca Kıykım, Betul Karaatmaca, Hulya Kose, Dilara Fatma Kocacık Uygun, Fatih Celmeli, Tugba Arikoglu, Dilek Ozcan, Ozlem Keskin, Elif Arık, Elif Soyak Aytekin, Mahmut Cesur, Ercan Kucukosmanoglu, Mehmet Kılıc, Mutlu Yuksek, Zafer Bıcakcı, Saliha Esenboga, Deniz Çagdaş Ayvaz, Asena Pınar Sefer, Sukrü Nail Guner, Sevgi Keles, Ismail Reisli, Ugur Musabak, Nazlı Deveci Demirbas, Sule Haskologlu, Sara Sebnem Kilic, Ayse Metin, Figen Dogu, Aydan Ikinciogulları, Ilhan Tezcan

**Affiliations:** 1 Institute of Child Health, Hacettepe University, Ankara, Turkey; 2 Can Sucak Research Laboratory for Translational Immunology, Hacettepe University, Ankara, Turkey; 3 Department of Pediatric Immunology, Institute of Child Health, Hacettepe University, Ankara, Turkey; 4 Pediatric Immunology, SBU Ankara Dr Sami Ulus Maternity Child Health and Diseases Training and Research Hospital, Ankara, Turkey; 5 Centre for Cancer Biology, University of South Australia and SA Pathology, Adelaide, SA 5000 Australia; 6 Department of Pediatric Allergy and Clinical Immunology, University of Health Sciences, Istanbul Basaksehir Cam and Sakura City Hospital, Istanbul, Turkey; 7 Department of Pediatric Allergy and Clinical Immunology, Altinbas University School of Medicine, Istanbul, Turkey; 8 Medical Park Bahçeşehir Hospital, Clinic of Hematology and Transplantation, İstanbul, Turkey; 9 Marmara University, Faculty of Medicine, Department of Pediatric Allergy and Immunology, Jeffrey Modell Diagnostic and Research Center for Primary Immunodeficiencies, The Isil Berat Barlan Center for Translational Medicine, Istanbul, Turkey; 10 Pediatric Allergy and Immunology, Cerrahpasa School of Medicine, Istanbul University-Cerrahpasa, Istanbul, Turkey; 11 Department of Pediatric Allergy and Immunology, University of Health Sciences, Ankara Bilkent City Hospital, Ankara, Turkey; 12 Department of Pediatric Immunology, Diyarbakir Children Hospital, Diyarbakır, Turkey; 13 Division of Allergy Immunology, Department of Pediatrics, Akdeniz University Faculty of Medicine, Antalya, Turkey; 14 Republic of Turkey Ministry of Health Antalya Training and Research Hospital Pediatric Immunology and Allergy Diseases, Antalya, Turkey; 15 Department of Pediatric Allergy and Immunology, Faculty of Medicine, Mersin University, Mersin, Turkey; 16 Division of Pediatric Allergy and Immunology, Faculty of Medicine, Balcali Hospital, Cukurova University, Adana, Turkey; 17 Department of Pediatric Allergy and Immunology, Faculty of Medicine, Gaziantep University, Gaziantep, Turkey; 18 Department of Pediatric Allergy and Immunology, Etlik City Hospital, Ankara, Turkey; 19 Division of Allergy and Immunology, Department of Pediatrics, Faculty of Medicine, University of Firat, Elazığ, Turkey; 20 Department of Pediatric Immunology and Allergy, Faculty of Medicine, Zonguldak Bulent Ecevit University, Zonguldak, Turkey; 21 Department of Pediatric Hematology, Faculty of Medicine, Ataturk University, Erzurum, Turkey; 22 Department of Pediatrics, Division of Pediatric Immunology, Hacettepe University School of Medicine, Ankara, Turkey; 23 Section of Pediatric Immunology, Institute of Child Health, Hacettepe University, Ankara, Turkey; 24 Department of Pediatric Allergy and Immunology, Şanlıurfa Training and Research Hospital, Şanlıurfa, Turkey; 25 Department of Pediatric Immunology and Allergy, Medicine Faculty, Necmettin Erbakan University, Konya, Turkey; 26 Department of Immunology and Allergy, Baskent University School of Medicine, Ankara, Turkey; 27 Department of Pediatric Immunology and Allergy, Ankara University Faculty of Medicine, Ankara, Turkey; 28 Division of Pediatric Immunology-Rheumatology, Bursa Uludag University Faculty of Medicine, Bursa, Turkey; 29 Translational Medicine, Bursa Uludag University, Bursa, Turkey; 30 Department of Pediatrics, Division of Pediatric Immunology, Hacettepe University School of Medicine, Ankara, Turkey

**Keywords:** Inborn errors of immunity, next generation sequencing, whole exome sequencing, genetic diagnosis

## Abstract

Molecular diagnosis of inborn errors of immunity (IEI) plays a critical role in determining patients’ long-term prognosis, treatment options, and genetic counseling. Over the past decade, the broader utilization of next-generation sequencing (NGS) techniques in both research and clinical settings has facilitated the evaluation of a significant proportion of patients for gene variants associated with IEI. In addition to its role in diagnosing known gene defects, the application of high-throughput techniques such as targeted, exome, and genome sequencing has led to the identification of novel disease-causing genes. However, the results obtained from these different methods can vary depending on disease phenotypes or patient characteristics. In this study, we conducted whole-exome sequencing (WES) in a sizable cohort of IEI patients, consisting of 303 individuals from 21 different clinical immunology centers in Türkiye. Our analysis resulted in likely genetic diagnoses for 41.1% of the patients (122 out of 297), revealing 52 novel variants and uncovering potential new IEI genes in six patients. The significance of understanding outcomes across various IEI cohorts cannot be overstated, and we believe that our findings will make a valuable contribution to the existing literature and foster collaborative research between clinicians and basic science researchers.

**Supplementary Information:**

The online version contains supplementary material available at 10.1007/s10875-024-01759-w.

## Introduction

Inborn errors of immunity or primary immunodeficiencies (PIDs) represent a diverse group of disorders characterized by increased susceptibility to infections, malignancy, allergy, and immune dysregulation [1]. While these diseases occur at a frequency of approximately 1 in 10,000 in the general population, their prevalence is higher in societies with elevated rates of consanguinity, such as Türkiye [2–4]. The genetic pleiotropy and heterogeneity observed in IEI contribute to the broad range of clinical manifestations associated with these disorders [5]. The majority of IEI cases are monogenic diseases with autosomal recessive inheritance patterns [5]. Therefore, comprehensive genetic diagnosis is vital for effective management of patients with IEI. In the past decade, NGS methods have revolutionized genetic screening, greatly enhancing the diagnostic capabilities for IEI [6]. This progress has led to an unprecedented increase in the identification of genes causing immunodeficiencies, with approximately 500 genetic defects associated with immunodeficiency currently recognized [7].

Founded in 2018 in memory of Can Sucak, who suffered from ZAP70 deficiency, the Candan Bişeyler Foundation (CSCBF) actively supports research in the field of IEI and raises awareness in Türkiye. The “Hacettepe University Can Sucak Research Laboratory for Translational Immunology” is dedicated to providing genetic diagnosis for immunodeficiency patients and conducting advanced functional research in a comprehensive manner throughout the country. This study presents the results of a comprehensive investigation into the genetic diagnosis of an extensive cohort of IEI patients from a specialized immune deficiency research center in Türkiye.

## Methods

### Study Participants

**Fig. 1:**
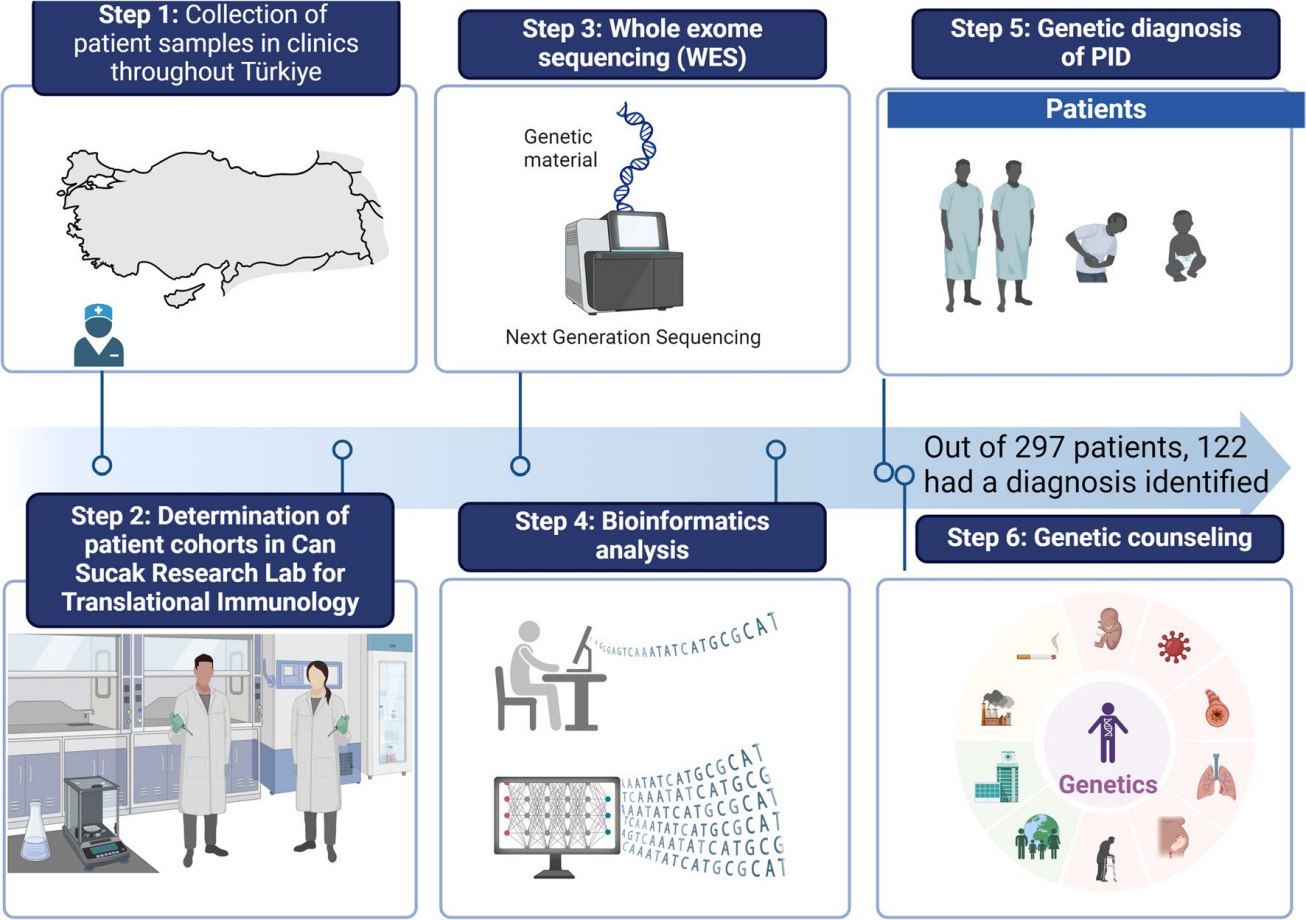
Schematic workflow of the study

Patients diagnosed with IEI based on clinical and laboratory characteristics between 2020 and 2023 were included in the study. These patients were recruited from multiple clinical immunology centers in Türkiye. Blood samples were collected from the patients following the guidelines and approval of the local Ethics Committee of Hacettepe University. Informed consent forms were obtained from the participants or their parents. The study's workflow is illustrated in Fig. 1.

### Whole Exome Sequencing and Variant Analysis

Genomic DNA was isolated from peripheral blood samples using a DNA isolation kit (GeneAll). The NGS exome library was prepared utilizing the Illumina Nextera DNA Prep with Enrichment Kit. Sequencing was carried out on the Illumina NextSeq 550 platform, generating 150-bp paired-end reads. Mapping, variant calling, and annotation were performed using SEQ Platform v8 (Genomize). Copy number variation (CNV) analysis was conducted using SEQ Platform as well.

To identify causative variants, we employed a filtering strategy that involved screening all variants identified from the WES data. Our focus was on exonic and splice site variants, excluding synonymous variants, and we specifically looked for rare variants with a minor allele frequency of less than 1% in different strategic gene groups. Initially, we examined rare variants in known IEI genes (approximately 500), followed by potential candidate genes predicted by the human gene connectome [8]. Finally, we assessed variants across the entire set of genes (Supplementary Figure 2A).

### Sanger Sequencing

To validate the identified variants, we conducted Sanger sequencing using standard protocols [9].

### RT-qPCR

RT-qPCR was utilized to validate the effects of structural variants. Total RNA was isolated from peripheral blood mononuclear cells (PBMCs) obtained from both patients and healthy controls using the NucleoSpin RNA Plus Kit (Macherey-Nagel). Subsequently, cDNA was synthesized using the iScript cDNA synthesis kit (Bio-Rad). RT-qPCR was carried out on the CFX Connect System (Bio-Rad) using the iTaq Universal SYBR Green Supermix (Bio-Rad) [10].

## Results

### Technical Output of the Sequencing Data

The results of the WES data showed a total number of reads ranging from 21.7 to 77.6 million (median: 46.1) (Supplementary Figure 2B). The average depth of coverage varied between 24.5 and 134.2 (median: 64.1) (Supplementary Figure 2C). The target regions (exons and splice regions) were covered at a depth of 20X from 89.02% to 99.91%, and at a depth of 50X from 68.13% to 99.65% (Supplementary Figure 2D).

### Patients

Our study involved a total of 303 individuals who were clinically diagnosed with IEI. These participants were recruited from 21 separate clinical immunology centers and they were selected after assessments with their clinicians. Especially, patients truly exhibited severe phenotypes of immunodeficiency were admitted to the study. However, six patients were excluded from the current analysis as they exhibited potential novel IEI-associated genes, pending further investigation through functional studies. Therefore, the analysis in this study includes 297 patients.

**Fig. 2:**
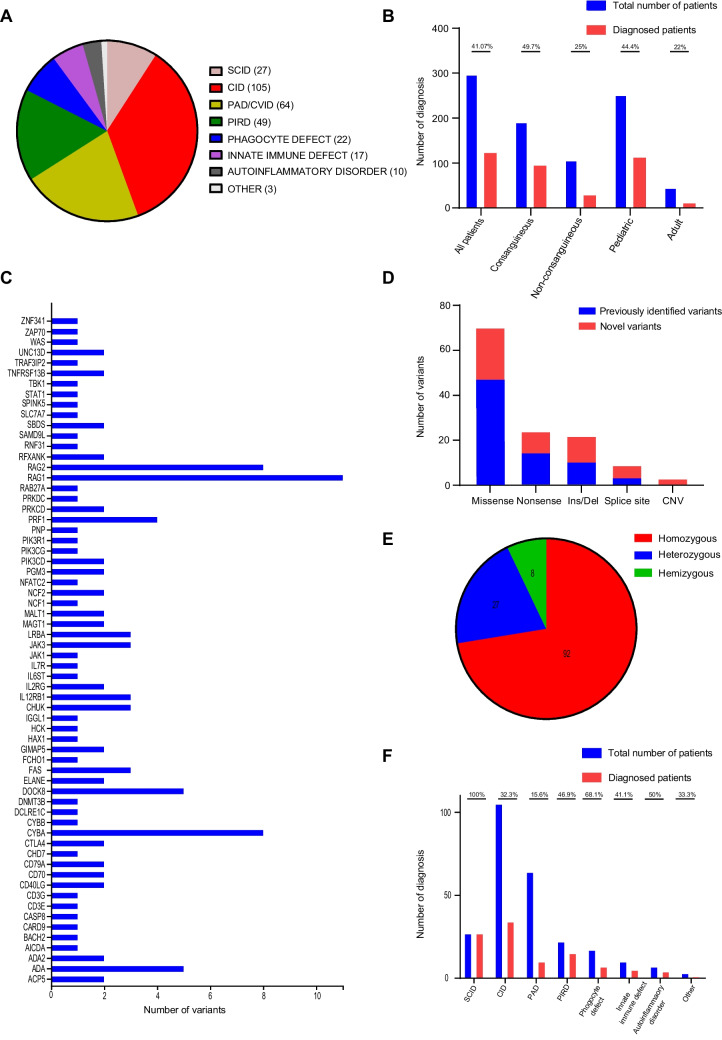
Patient and variant characteristics. **A** Distribution of the patients based on their clinical diagnosis. **B** Diagnostic yield of the patients. **C** Number of the detected variants and their distribution across different IEI genes. **D** Types of detected variants and their novelty. **E** Distribution of zygosity. **F** Number of diagnosis in patient groups

Among the included patients, there were 145 males and 152 females, representing a relatively balanced gender distribution. The age range of the participants varied from three months to 42 years, with a median age of nine years. The majority of the cohort consisted of pediatric patients (n=252), while a smaller subset comprised adult patients (n=45). A notable observation in our study was the high consanguinity rate, with 64.6% (192 out of 297 cases) of patients demonstrating consanguineous relationships within their families. The distribution of clinical diagnoses, classified according to the International Union of Immunological Societies (IUIS) classification, included 27 cases of Severe Combined Immunodeficiency (SCID), 105 cases of Combined Immunodeficiency (CID), 64 cases of Primary Antibody Deficiency (PAD), 49 cases of Primary Immune Regulatory Disorder (PIRD), 22 cases of congenital anomalies affecting phagocyte number/function, 17 cases of disorders of intrinsic and innate immunity, 10 cases of autoinflammatory disorders, and 3 cases of other classified IEI. These other cases potentially involve bone marrow failure or complement deficiencies, as illustrated in Fig. 2A.

### Results of Genetic Diagnosis and the Profile of Disease-Causing Variants

In our cohort, a genetic diagnosis was established in 122 out of the 297 patients examined, with a total of 127 potential genetic variants identified. This yielded a diagnostic rate of 41.1%. Among the 193 patients with consanguineous parents, causative genetic defects were identified in 95 individuals, resulting in a diagnostic rate of 49.7%. On the other hand, among the 106 patients from non-consanguineous parents, 28 individuals (25.7%) received a genetic diagnosis. The diagnostic rate was higher in pediatric patients, with 44.4% (112 out of 252) receiving a genetic diagnosis, compared to the adult group, which had a lower rate of 22% (10 out of 45) (Fig. 2B). Details of all identified genetic variants and their associated clinical features are presented in Table 1, Table 2 and Supplementary Table 3. In addition, variant characteristics including American College of Medical Genetics (ACMG) criteria and pathogenicity prediction scores were given in Supplementary Table 1). Overall, a total of 127 likely causative genetic anomalies were identified across 64 known IEI genes, as depicted in Fig. 2C. Among these genetic variants, 75 had been previously reported in public databases, while 52 were novel findings reported in this study (Fig. 2D). The variants consisted of 92 homozygous, 27 heterozygous, and 8 hemizygous mutations (Fig. 2E). The spectrum of variant types included 69 missense mutations, 24 nonsense mutations, 22 insertion/deletions (indels), 9 essential splice site variations, and 3 copy number variations (Figure 2D). CNV analysis was performed on 57 subjects using a strategy that incorporated samples with comparable mean read depths. The implications of the CNVs were validated through capillary sequencing or quantitative PCR (qPCR). The causality of monoallelic variants was evaluated based on clinical and laboratory features of the patients, literature associations, or different functional analyses (Supplementary Table 2). The diagnostic rates across different disease categories were as follows: Severe Combined Immunodeficiency (SCID) had a diagnostic rate of 100%, congenital anomalies affecting phagocyte number/function at 68.1%, autoinflammatory disorders at 50%, Primary Immune Regulatory Disorder (PIRD) at 46.9%, intrinsic and innate immunity defects at 41.1%, Combined Immunodeficiency (CID) at 32.3%, other forms of IEI at 33.3%, and Primary Antibody Deficiency (PAD) at 15.6%, and (Fig. 2F).

**Table 1:** Details of the variants detected in the study

Patient no	Clinical diagnosis (IUIS)	Age	Gender	Consan.	Gene	Variant	Transcript ID	Zygosity	Consequence	Novelty
P1 [11]	Innate immune defect	9	M	+	*CARD9*	c.883C>T p.Gln295Ter	NM_052813.4	Hom	Nonsense	rs1833232307
P2 [12]	CID	6	M	+	*RFXANK*	c.634C>T p.Arg212Ter	NM_003721.3	Hom	Nonsense	rs747402973
P3 [13]	SCID	7	M	+	*CD3E*	c.176G>A p.Trp59Ter	NM_000733.3	Hom	Nonsense	rs121918659
P4	CID	12	F	+	*NFATC2*	c.340_345delGAGATC p.Glu114_Ile115del	NM_173091.3	Hom	Inframe Deletion	Novel
P5 [12]	SCID	6 m	F	+	*JAK3*	c.2134G>A p.Gly712Ser	NM_000215.4	Hom	Missense	rs1178958564
P6 [12]	SCID	8 m	F	+	*RAG2*	c.581C>A p.Ser194Ter	NM_000536.3	Hom	Nonsense	Novel
P7 [12]	SCID	2	M	+	*RAG1*	c.2005G>A p.Glu669Lys c.1307C>A p.Thr436Asn	NM_000448.3 NM_000448.2	Comp. Het	Missense Missense	rs878853004 Novel
P8 [12]	SCID	1	M	+	*RAG1*	c.2005G>A p.Glu669Lys c.1307C>A p.Thr436Asn	NM_000448.2	Comp. Het	Missense Missense	rs878853004 Novel
P9 [14]	PIRD	6	M	+	*CD70*	c.332C>T p.Thr111Met	NM_001252.3	Hom	Missense	rs1378830614
P10 [14]	PIRD	4	M	+	*CD70*	c.332C>T p.Thr111Met	NM_001252.3	Hom	Missense	rs1378830614
P11 [12]	Phagocyte defect	9	F	+	*CYBA*	c.58+4_58+7delAGTG	NM_000101.4	Hom	Splice site/Deletion	rs771926427
P12 [15]	CID	6	M	+	*ZNF341*	c.1626C>G p.Tyr542Ter	NM_001282933.2	Hom	Nonsense	rs376598954
P13	CID	7	F	+	*ZAP70*	c.1010T>G p.Leu337Ala	NM_001079.4	Hom	Missense	rs1254428002
P14 [16, 17]	SCID	3 m	M	+	*RAG2*	c.105G>C p.Gly35Ala	NM_000536.4	Hom	Missense	rs148508754
P15 [16, 17]	SCID	1	M	+	*RAG2*	c.105G>C p.Gly35Ala	NM_000536.4	Hom	Missense	rs148508754
P16 [18, 19]	PAD/CVID	40	F	+	*TNFRSF13B*	c.310C>T p.Cys104Arg	NM_012452.3	Hom	Missense	rs34557412
P17	PAD/CVID	3	F	+	*PIK3R1*	c.837-1G>A	NM_181523.2	Hom	Splice site/Missense	Novel
P18	CID	20	F	+	*PGM3*	c.214G>A p.Gly72Ser	NM_001199919.1	Hom	Missense	Novel
P19	Other	2	F	-	*SAMD9L*	c.2639A>C p.His880Pro	NM_001350083	Het	Missense	Novel
P20 [18]	PAD/CVID	17	M	+	*TNFRSF13B*	c.204dupA p.Leu69Tfs*11	NM_012452.3	Hom	Out of frame/Insertion	rs72553875
P21	PAD/CVID	24	F	+	*CD79A*	c.380-2A>G	NM_001783	Hom	Splice Site/Missense	Novel
P22	CID	34	F	+	*DNMT3B*	c.2029G>A p.Val677Met	NM_006892.4	Hom	Missense	rs866792483
P23	PAD/CVID	34	F	+	*AICDA*	c.A100T p.Lys34Ter	NM_001330343	Hom	Nonsense	Novel
P24 [20, 21]	Phagocyte defect	2	F	+	*CYBA*	c.G70A p.Gly24Arg	NM_000101.4	Hom	Missense	rs28941476
P25 [22]	CID	13	M	+	*MALT1*	c.1318_1321delTGTC p.L440Valfs*6	NM_006785.4	Hom	Out of frame/Deletion	rs140664950
P26	Phagocyte defect	10	F	-	*SBDS*	c.578T>C p.Lys193Pro c.184A>T p.Lys62Ter	NM_016038.4	Comp. Het	Missense Nonsense	rs120074160 rs1195681400
P27	CID	10	M	+	*RFXANK*	Exon 2-6 Deletion	NM_003721.3	Hom	CNV	Novel
P28	PIRD	11	F	-	*MAGT1*	c.199-16A>G	NM_032121.5	Hem	Splice Site/Missense	Novel
P29	SCID	6 m	F	+	*ADA*	c.551_555del p.Glu184Glyfs*2 c.241G>A p.Gly81Arg	NM_001322050 NM_001322050	Comp. Het	Out of frame/Deletion Missense	Novel rs2065384316
P30	SCID	1	F	+	*RAG1*	c.1767C>G p.Tyr589Ter	NM_000448.2	Hom	Nonsense	Novel
P31	SCID	8 m	F	+	*JAK3*	c.932delC p.Pro311Argfs*17	NM_000215	Hom	Out of frame/Deletion	Novel
P32	Innate immune defect	2	M	+	*TRAF3IP2*	c.559C>T p.Arg187Ter	NM_147686.3	Hom	Nonsense	rs762395569
P33	SCID	9 m	M	+	*RAG1*	c.2126G>A p.Gly709Asp	NM_000448.2	Hom	Missense	Novel
P34	SCID	1	M	+	*ADA*	c.779A>G p.Glu260Gly	NM_000022.4	Hom	Missense	rs1354071013
P35	Phagocyte defect	10	M	+	*NCF2*	c.233G>A p.Gly78Glu	NM_000433.4	Hom	Missense	rs137854519
P36	Phagocyte defect	1	F	+	*CYBA*	c.166dupC p.Arg56Profs*156	NM_000101	Hom	Out of frame/Insertion	rs1555550793
P37	PIRD	9	M	+	*LRBA*	c.646-1G>A	NM_006726.4	Hom	Splice site/Missense	rs1741243666
P38	SCID	10 m	F	+	*JAK3*	c.2080G>T p.Glu694Ter	NM_000215.3	Hom	Nonsense	Novel
P39	SCID	4	M	-	*IL2RG*	c.437T>A p.Leu146Gln	NM_000206.2	Hem	Missense	Novel
P40	PIRD	19	M	+	*PRKCD*	c.1097G>A p.Gly366Glu	NM_001354680.2	Hom	Missense	Novel
P41	SCID	1	M	+	*RAG2*	c.623T>A p.Val208Asp	NM_001243786.1	Hom	Missense	Novel
P42 [23–26]	PIRD	15	M	-	*CTLA4*	c.118G>A p.Val40Met	NM_005214.5	Het	Missense	rs1553657378
P43	PIRD	17	M	-	*JAK1*	c.2485A>G p.Asn829Asp	NM_001321853.2	Het	Missense	Novel
P44	SCID	9 m	F	+	*RAG1*	c.1767C>G p.Tyr589Ter	NM_000448.2	Hom	Nonsense	Novel
P45	PIRD	18	M	+	*PRKCD*	c.1097G>A p.Gly366Glu	NM_001354680.2	Hom	Missense	Novel
P46	Phagocyte defect	32	M	-	*CYBB*	c.770G>A p.Cys257Tyr	NM_000397.4	Hem	Missense	Novel
P47	CID	8	F	+	*CHUK*	c.499G>A p.Gly167Arg	NM_001278.5	Hom	Missense	Novel
P48	CID	4	F	+	*CHUK*	c.499G>A p.Gly167Arg	NM_001278.5	Hom	Missense	Novel
P49	SCID	1	F	+	*RAG1*	c.742C>T p.Gln248Ter	NM_000448.2	Hom	Nonsense	Novel
P50	CID	10	M	-	*CD40L*	c.15C>A p.Tyr5Ter	NM_000074.3	Hem	Nonsense	Novel
P51	PIRD	1	M	+	*UNC13D*	c.2346_2349delGGAG p.Arg782SerfsTer12	NM_199242.2	Hom	Out of frame/Deletion	rs764196809
P52 [27]	PAD/CVID	2	F	+	*IGGL1*	c.425C>T p.Pro142Leu	NM_020070.4	Hom	Missense	rs1064422
P53	Phagocyte defect	1	F	-	*ELANE*	c.703delG p.Val235TrpfsTer5	NM_001972.4	Het	Out of frame/Deletion	Novel
P54	Autoinflammatory disorder	42	F	-	*HCK*	c.135_136delinsTG p.Pro46Ala	NM_002110.4	Het	Indel	Novel
P55	Phagocyte defect	11	M	+	*CYBA*	c.385G>A p.Glu129Lys	NM_000101.4	Hom	Missense	rs1246768740
P56	PIRD	17	M	+	*SLC7A7*	c.1417C>T p.Arg473Ter	NM_001126106.2	Hom	Nonsense	rs386833808
P57 [28, 29]	Phagocyte defect	4	M	+	*NCF2*	c.196C>T p.Arg66Ter	NM_000433.3	Hom	Nonsense	rs750782115
P58	SCID	2	M	+	*DCLRE1C*	c.1633del p.Glu545Asnfs*58	NM_001350965.2	Hom	Out of frame/Deletion	Novel
P59	SCID	8 m	F	+	*RAG2*	c.712delC p.Val238LeufsTer10	NM_001243786.1	Hom	Out of frame/Deletion	Novel
P60 [30, 31]	Innate immune defect	18	F	+	*IL12RB1*	c.523C>T p.Arg175Trp	NM_005535.3	Hom	Missense	rs750667928
P61	CID	12	M	+	*CD40L*	c.15C>A p.Tyr5Ter	NM_000074.3	Hom	Nonsense	Novel
P62 [32–34]	Autoinflammatory disorder	15	M	+	*ADA2*	c.1072G>A p.Gly358Arg	NM_001282225.2	Hom	Missense	rs45511697
P63 [35, 36]	Innate immune defect	2	M	+	*IL12RB1*	c.1456C>T p.Arg486Ter	NM_005535.3	Hom	Nonsense	rs576374797
P64	CID	2	F	+	*CHUK*	c.499G>A p.Gly167Arg	NM_000074.3	Hom	Missense	Novel
P65	Phagocyte defect	6	M	+	*CYBA*	c.371C>T p.Ala124Val	NM_000101.4	Hom	Missense	rs179363894
P66 [37]	CID	17	M	+	*GIMAP5*	c.667C>T p.Leu223Phe	NM_018384.5	Hom	Missense	rs2116581086
P67 [37]	CID	12	F	+	*GIMAP5*	c.667C>T p.Leu223Phe	NM_018384.5	Hom	Missense	rs2116581086
P68	PAD/CVID	7	F	+	*CD79A*	c.177dup p.Asn60GlnfsTer20	NM_001783.4	Hom	Out of frame/Insertion	Novel
P69	PIRD	1	F	+	*UNC13D*	c.1082del p.Tyr361SerfsTer43	NM_199242.2	Hom	Out of frame/Deletion	Novel
P70 [38]	PIRD	19	M	-	*FAS*	c.361C>T p.Arg121Trp	NM_000043.6	Het	Missense	rs121913078
P71 [39, 40]	PIRD	1	F	+	*PRF1*	c.1122G>A p.Trp374Ter	NM_005041.5	Hom	Nonsense	rs104894176
P72	CID	6	M	+	*DOCK8*	c.5831C>T p.Pro1944Leu	NM_203447.3	Hom	Missense	rs775779897
P73	CID	4	F	+	*DOCK8*	c.5831C>T p.Pro1944Leu	NM_203447.3	Hom	Missense	rs775779897
P74 [26, 41]	PIRD	14	F	-	*CTLA4*	c.151C>T p.Arg51Ter	NM_005214.5	Het	Nonsense	rs606231417
P75 [42, 43]	Phagocyte defect	5	M	+	*HAX1*	c.130_131insA p.Trp44Ter	NM_006118.4	Hom	Out of frame	rs1572018284
P76	CID	6	F	+	*PIK3CG*	c.2159A>G p.Tyr720Cys	NM_002649.3	Hom	Missense	rs199590448
P77	CID	7	M	+	*MALT1*	c.1133T>G p.Phe378Cys	NM_006785.4	Hom	Missense	novel
P78	PIRD	12	M	-	*MAGT1*	c.628-4T>C	NM_032121.5	Hem	Splice site/Missense	novel
P79 [44]	Autoinflammatory disorder	17	M	+	*ACP5*	c.772_790del p.Ser258WTrpfs*39	NM_001322023.2	Hom	Out of frame/Deletion	rs878853218
P80 [45]	CID	1	F	+	*PGM3*	c.821A>G p.Asn274Ser	NM_001199917.2	Hom	Missense	rs587777562
P81 [46]	CID	9	F	+	*CD3G*	c.80-1G>C	NM_000073.2	Hom	Splice site/Missense	rs775848095
P82	Phagocyte defect	2	M	-	*ELANE*	c.367-8C>A	NM_001972.4	Het	Splice site/Missense	novel
P83 [20, 47]	Phagocyte defect	16	F	-	*CYBA*	c.70G>A p.Gly24Arg c.373G>A p.Ala125Thr	NM_000101.4 NM_000101.4	Comp. Het	Missense Missense	rs28941476 rs119103269
P84	Autoinflammatory disorder	16	M	+	*ADA2*	c.319A>C p.Lys107Gln	NM_001282225.2	Hom	Missense	novel
P85	PIRD	6	M	-	*FAS*	c.761T>A p.Val254Asp	NM_000043.6	Het	Missense	novel
P86	CID	3	F	+	*PNP*	c.461+1G>A	NM_000270.3	Hom	Splice site/Missense	novel
P87 [48–51]	PIRD	9	M	+	*RAB27A*	c.514_518del p.Gln172AsnfsTer2	NM_004580.5	Hom	Out of frame/Deletion	rs767481076
P88	CID	16	M	-	*BACH2*	c.745del p.Ser249ValfsTer93	NM_021813.2	Het	Out of frame/Deletion	novel
P89	CID	6	M	+	*RNF31*	c.2846A>C p.Asn949Thr	NM_017999.5	Hom	Missense	rs766565788
P90 [44]	Autoinflammatory disorder	2	F	+	*ACP5*	c.772_790del Ser258Trpfs*39	NM_001322023.2	Hom	Out of frame/Deletion	rs878853218
P91 [39, 40]	PIRD	2	F	+	*PRF1*	c.1122G>A p.Trp374Ter	NM_005041.5	Hom	Nonsense	rs104894176
P92	Phagocyte defect	14	F	+	*NCF1*	Exon 5-6 Dup	NM_000265	Hom	CNV	novel
P93	CID	2	M	-	*CHD7*	c.1904A>T p.Asp635Val	NM_017780.4	Het	Missense	rs752468864
P94	CID	17	M	+	*FCHO1*	c.2183A>C p.Asn728Thr	NM_001161357.1	Hom	Missense	novel
P95 [52–54]	PIRD	4	M	+	*LRBA*	c.2836_2839del p.Glu946Ter	NM_006726.4	Hom	Out of frame/Deletion	rs777413769
P96	Innate immune defect	8	M	-	*TBK1*	c.1055T>C p.Leu352Pro	NM_013254.4	Het	Missense	novel
P97	SCID	1	M	+	*IL7R*	c.337G>T p.Glu113Ter	NM_002185.5	Hom	Nonsense	novel
P98 [52–54]	PIRD	20	F	+	*LRBA*	c.2836_2839del p.Glu946Ter	NM_006726.4	Hom	Out of frame/Deletion	rs777413769
P99 [55–57]	SCID	9 m	F	+	*PRKDC*	c.9182T>G p.Leu3061Arg	NM_006904.7	Hom	Missense	rs587777685
P100 [58, 59]	SCID	16	F	+	*RAG2*	c.104G>C p.Gly35Ala	NM_001243786.1	Hom	Missense	rs148508754
P101 [60, 61]	PAD/CVID	6	F	-	*PIK3CD*	c.1573G>A p.Glu525Lys	NM_005026.5	Het	Missense	rs587777389
P102	PIRD	14	M	-	*FAS*	c.340G>A p.Glu114Lys	NM_000043.6	Het	Missense	rs773565107
P103	Innate immune defect	11	F	-	*STAT1*	c.1192G>A p.Gly397Ser	NM_007315.3	Het	Missense	novel
P104	CID	12	F	-	*IL6ST*	c.2093C>A p.Ala698Glu	NM_002184.4	Het	Missense	rs745818447
P105 [62, 63]	Innate immune defect	10	M	+	*IL12RB1*	c.637C>T p.Arg213Trp	NM_005535.3	Hom	Missense	rs121434494
P106	CID	2	F	+	*DOCK8*	c.5766G>A p.Met1922Ile	NM_203447.4	Hom	Missense	rs2057267200
P107	CID	1	F	+	*DOCK8*	Exon 1-10 Deletion	NM_203447.4	Hom	CNV	novel
P108	CID	5	M	+	*SPINK5*	c.2658_2662dupGAGCA p.Ile888ArgfsTer56	NM_001127698.1	Hom	Out of frame/Dup	novel
P109 [64]	SCID	6 m	M	+	*ADA*	c.556G>A p.Glu186Lys	NM_000022.4	Hom	Missense	rs1555844416
P110 [65–67]	CID	2	M	+	*RAG1*	c.2095C>T p.Arg699Trp	NM_000448.3	Hom	Missense	rs199474676
P111	PIRD	3 m	M	+	*PRF1*	c.1267delC p.Gln423LysfsX17	NM_005041.5	Hom	Out of frame/Deletion	novel
P112	SCID	3 m	M	+	*IL2RG*	c.511G>T p.Glu171Ter	NM_000206.2	Hem	Nonsense	novel
P113 [68, 69]	PAD/CVID	7	F	+	*CASP8*	c.919C>T p.Arg307Trp	be NM_001080125.1	Hom	Missense	rs17860424
P114	CID	18	F	+	*DOCK8*	c.5831C>T p.Pro1944Leu	NM_203447.4	Hom	Missense	rs775779897
P115 [64]	SCID	9 m	M	+	*ADA*	c.556G>A p.Glu186Lys	NM_000022.4	Hom	Missense	rs1555844416
P116	SCID	1	F	+	*RAG1*	c.1307C>A p.Thr436Asn	NM_000448.2	Hom	Missense	novel
P117 [29, 70, 71]	SCID	1	F	+	*RAG1*	c.2210G>A p.Arg737His	NM_000448.3	Hom	Missense	rs104894286
P118 [20]	Phagocyte defect	5	F	+	*CYBA*	c.70G>A p.Gly24Arg	NM_000101.4	Hom	Missense	rs28941476
P119	PIRD	3	F	+	*PRF1*	c.1385C>A p.Ser462Ter	NM_005041.5	Hom	Nonsense	rs1564723653
P120	CID	4	M	-	*WAS*	c.37C>T p.Arg13Ter	NM_000377.3	Hem	Nonsense	rs193922415
P121	CID	5	M	-	*WAS*	c.91G>A p.Glu31Lys	NM_000377.3	Hem	Missense	rs1557006239
P122	PAD/CVID	9	M	-	*PIK3CD*	c.1573G>A p.Glu525Lys	NM_005026.5	Het	Missense	rs587777389

*SCID* Severe combined immunodeficiency, *CID* Combined immunodeficiency, *PAD* Primary antibody deficiency, *CVID* Common variable immunodeficiency, *PIRD* Primary immune regulation disorder, *m* months, *M* Male, *F* Female, *Consan* Consanguinity, *Hom* Homozygous, *Het* Heterozygous, *Hem* Hemizygous, *CNV* Copy number variation

**Table 2:** Clinical features of the patients associated with detected gene defects

Patient no	Clinical diagnosis (IUIS classification)	Gene	Variant	Associated features of the patients
P1	Innate immune defect	*CARD9*	c.883C>T p.Gln295Ter	Invasive fungal infection, HSM, dermatitis, elevated IgG and IgE
P2	CID	*RFXANK*	c.634C>T p.Arg212Ter	Failure to thrive, respiratory and gastrointestinal infections, low CD4+ T cells
P3	SCID	*CD3E*	c.176G>A p.Trp59Ter	T - B+ NK+
P4	CID	*NFATC2*	c.340_345delGAGATC p.Glu114_Ile115del	EBV-associated lymphoproliferation, recurrent pulmonary infections, hypogammaglobulinemia
P5	SCID	*JAK3*	c.2134G>A p.Gly712Ser	T - B+ NK+
P6	SCID	*RAG2*	c.581C>A p.Ser194Ter	T - B- NK+
P7	SCID	*RAG1*	c.2005G>A p.Glu669Lys c.1307C>A p.Thr436Asn	T - B- NK+
P8	SCID	*RAG1*	c.2005G>A p.Glu669Lys c.1307C>A p.Thr436Asn	T - B- NK+
P9	PIRD	*CD70*	c.332C>T p.Thr111Met	Burkitt lymphoma, hypogammaglobulinemia, reduced memory B cells
P10	PIRD	*CD70*	c.332C>T p.Thr111Met	Recurrent pulmonary infections, non-Hodgkin lymphoma, hypogammaglobulinemia
P11	Phagocyte defect	*CYBA*	c.58+4_58+7delAGTG	Pulmonary Aspergillus infections, lymphadenitis, defective oxidative burst
P12	CID	*ZNF341*	c.1626C>G p.Tyr542Ter	Early onset eczema, recurrent skin and pulmonary infections, eosinophilia, elevated IgE
P13	CID	*ZAP70*	c.1010T>G p.Leu337Ala	CMV infection, chronic diarrhea, recurrent bacterial infections, low CD8+ T cells
P14	SCID	*RAG2*	c.105G>C p.Gly35Ala	T - B- NK+
P15	SCID	*RAG2*	c.105G>C p.Gly35Ala	T - B- NK+
P16	PAD/CVID	*TNFRSF13B*	c.T310C p.Cys104Arg	Recurrent pulmonary infections, ITP, panhypogammaglobulinemia, reduced switched memory B cells
P17	PAD/CVID	*PIK3R1*	c.837-1G>A	Recurrent pulmonary infections, septic arthritis, agammaglobulinemia
P18	CID	*PGM3*	c.G214A p.Gly72Ser	Severe atopy, bacterial and viral infections, scoliosis, achondroplasia, dysgerminoma, reduced B and memory B cells, elevated IgE
P19	Other	*SAMD9L*	c.A2639C p.His880Pro	Aplastic anemia, recurrent bacterial infections, agammaglobulinemia, reduced NK cells
P20	PAD/CVID	*TNFRSF13B*	c.204dupA p.Leu69Tfs*11	Lichen planus, panhypogammaglobulinemia
P21	PAD/CVID	*CD79A*	c.380-2A>G	IBD, recurrent diarrhea, agammaglobulinemia, undetectable CD19+ B cells
P22	CID	*DNMT3B*	c.G2029A p.Val677Met	Recurrent pulmonary infections, osteoporosis, agammaglobulinemia, reduced T and B cells
P23	PAD/CVID	*AICDA*	c.A100T p.Lys34Ter	Rheumatoid arthritis, bacterial infections, elevated IgM
P24	Phagocyte defect	*CYBA*	c.G70A p.Gly24Arg	BCGitis, anal and liver abscess, defective oxidative burst
P25	CID	*MALT1*	c.1318_1321delTGTC p.L440Valfs*6	Bacterial, viral, fungal infections, defective T cell proliferation
P26	Phagocyte defect	*SBDS*	c.T578C p.Lys193Pro c.A184T p.Lys62Ter	Recurrent sinopulmonary infections, gingivitis, neutropenia
P27	CID	*RFXANK*	Exon 2-6 Deletion	Failure to thrive, recurrent sinopulmonary and gastrointestinal infections, warts, low CD4+ T cells
P28	PIRD	*MAGT1*	c.199-16A>G	EBV infection, lymphoma, hypogammaglobulinemia, decreased memory B cells
P29	SCID	*ADA*	c.551_555del p.Glu184Glyfs*2 c.G241A p.Gly81Arg	T - B- NK-
P30	SCID	*RAG1*	c.C1767G p.Tyr589Ter	T - B- NK+
P31	SCID	*JAK3*	c.932delC p.Pro311Argfs*17	T - B+ NK-
P32	Innate immune defect	*TRAF3IP2*	c.C559T p.Arg187Ter	CMC, alopecia areata, skin rashes
P33	SCID	*RAG1*	c.G2126A p.Gly709Asp	T - B- NK+
P34	SCID	*ADA*	c.A779G p.Glu260Gly	T - B- NK-
P35	Phagocyte defect	*NCF2*	c.G233A p.Gly78Glu	Recurrent infections, aphthous stomatitis, cervical lymphadenitis, occasional skin infections, defective oxidative burst
P36	Phagocyte defect	*CYBA*	c.166dupC p.Arg56Profs*156	Recurrent infections, cervical lymphadenitis, defective oxidative burst
P37	PIRD	*LRBA*	c.646-1G>A	AIHA, HSM, hypogammaglobulinemia, slightly decreased CD4+ T cells
P38	SCID	*JAK3*	c.G2080T p.Glu694Ter	T - B+ NK-
P39	SCID	*IL2RG*	c.437T>A p.Leu146Gln	T - B+ NK-
P40	PIRD	*PRKCD*	c.1097G>A p.Gly366Glu	BCGosis, meningitis, lymphoproliferation, CGD-like presentation
P41	SCID	*RAG2*	c.623T>A p.Val208Asp	T - B- NK+
P42	PIRD	*CTLA4*	c.118G>A p.Val40Met	AIHA, enteropathy, reduced T and B cells
P43	PIRD	*JAK1*	c.2485A>G p.Asn829Asp	IBD, lymphopenia, vitiligo, recurrent diarrhea, lymphopenia
P44	SCID	*RAG1*	c.C1767G p.Tyr589Ter	T - B- NK+
P45	PIRD	*PRKCD*	c.1097G>A p.Gly366Glu	SLE, thrombocytopenia, failure to thrive, skin rashes, mental retardation, hypogammaglobulinemia
P46	Phagocyte defect	*CYBB*	c.770G>A p.Cys257Tyr	Lymphoproliferation, granulomatous hepatitis, cytopenia, defective oxidative burst
P47	CID	*CHUK*	c.499G>A p.Gly167Arg	Recurrent bacterial, viral, fungal infections, chronic diarrhea, failure to thrive, hepatic fibrosis, absent secondary lymphoid tissues, hypogammaglobulinemia, reduced switched memory B cells
P48	CID	*CHUK*	c.499G>A p.Gly167Arg	Recurrent bacterial, viral, fungal infections, chronic diarrhea, failure to thrive, absent secondary lymphoid tissues, hypogammaglobulinemia, reduced switched memory B cells
P49	SCID	*RAG1*	c.742C>T p.Gln248Ter	T - B- NK+
P50	CID	*CD40L*	c.15C>A p.Tyr5Ter	Recurrent sinopulmonary infections, hypereosinophilia, eosinophilic gastroenteritis, memory B cells absent
P51	PIRD	*UNC13D*	c.2346_2349delGGAG p.Arg782SerfsTer12	HLH, pancytopenia, reduced naive T and RTE cells
P52	PAD/CVID	*IGGL1*	c.425C>T p.Pro142Leu	Recurrent bacterial, viral, fungal infections, panhypogammaglobulinemia
P53	Phagocyte defect	*ELANE*	c.703delG p.Val235TrpfsTer5	Recurrent bacterial infections, severe congenital neutropenia
P54	Autoinflammatory disorder	*HCK*	c.135_136delinsTG p.Pro46Ala	Nodulocystic acnes, cutaneous vasculitis, HSM
P55	Phagocyte defect	*CYBA*	c.385G>A p.Glu129Lys	Lung granulomas, chronic diarrhea, defective oxidative burst
P56	PIRD	*SLC7A7*	c.1417C>T p.Arg473Ter	Mental motor retardation, failure to thrive, skeletal anomalies, acanthosis nigricans, AIHA, lymphopenia
P57	Phagocyte defect	*NCF2*	c.196C>T p.Arg66Ter	Recurrent bacterial, fungal infections, lung granulomas, defective oxidative burst
P58	SCID	*DCLRE1C*	c.1633delT p.Glu545AsnfsTer	T - B- NK+
P59	SCID	*RAG1*	c.712delC p.Val238LeufsTer10	T - B- NK+
P60	Innate immune defect	*IL12RB1*	c.523C>T p.Arg175Trp	BCGitis
P61	CID	*CD40L*	c.15C>A p.Tyr5Ter	Asymptomatic, reduced switched memory B cells
P62	Autoinflammatory disorder	*ADA2*	c.1072G>A p.Gly358Arg	Recurrent pulmonary infections, reduced switched memory B and marginal zone B cells
P63	Innate immune defect	*IL12RB1*	c.1456C>T p.Arg486Ter	BCGitis, BCG lymphadenitis
P64	CID	*CHUK*	c.499G>A p.Gly167Arg	Recurrent pulmonary infections, absent secondary lymphoid tissues, hypogammaglobulinemia, reduced switched memory B cells
P65	Phagocyte defect	*CYBA*	c.371C>T p.Ala124Val	Recurrent sinopulmonary infections, recurrent fungal infections, deafness, defective oxidative burst
P66	CID	*GIMAP5*	c.667C>T p.Leu223Phe	Hodgkin lymphoma
P67	CID	*GIMAP5*	c.667C>T p.Leu223Phe	Hodgkin lymphoma
P68	PAD/CVID	*CD79A*	c.177dup p.Asn60GlnfsTer20	Chronic diarrhea, elevated hepatic transaminases, failure to thrive, agammaglobulinemia
P69	PIRD	*UNC13D*	c.1082del p.Tyr361SerfsTer43	HLH, pancytopenia
P70	PIRD	*FAS*	c.361C>T p.Arg121Trp	Splenomegaly, lymphadenopathy, ITP
P71	PIRD	*PRF1*	c.1122G>A p.Trp374Ter	HLH, HSM, reduced NK cells
P72	CID	*DOCK8*	c.5831C>T p.Pro1944Leu	Human papillomavirus (HPV) infections, recurrent sinopulmonary and gastrointestinal infections, elevated IgE, reduced naive and increased memory CD8+ T cells
P73	CID	*DOCK8*	c.5831C>T p.Pro1944Leu	Recurrent sinopulmonary and gastrointestinal infections, severe atopy, eosinophilia, elevated IgE, reduced naive and increased memory CD8+ T cells
P74	PIRD	*CTLA4*	c.151C>T p.Arg51Ter	Lymphadenopathy, lymphopenia, hypogammaglobulinemia, reduced switched memory B cells
P75	Phagocyte defect	*HAX1*	c.130_131insA p.Trp44Ter	Recurrent perianal abscess, neutropenia
P76	CID	*PIK3CG*	c.2159A>G p.Tyr720Cys	Severe atopic dermatitis, multiple food allergies, eosinophilia, hypogammaglobulinemia
P77	CID	*MALT1*	c.1133T>G p.Phe378Cys	Failure to thrive, moniliasis, necrotizing skin lesions, lymphoproliferation
P78	PIRD	*MAGT1*	c.628-4T>C	Recurrent sinopulmonary infections, wet cough, panhypogammaglobulinemia
P79	Autoinflammatory disorder	*ACP5*	c.772_790del p.Ser258WTrpfs*39	B-ALL, failure to thrive, spondyloenchondrodysplasia, intracranial calcification, mild MR
P80	CID	*PGM3*	c.821A>G p.Asn274Ser	Facial dysmorphic features, pancytopenia, T cell lymphopenia, reduced T lymphocyte activation
P81	CID	*CD3G*	c.80-1G>C	Recurrent sinopulmonary infections, AIHA, panhypogammaglobulinemia, reduced memory and switched memory B cells
P82	Phagocyte defect	*ELANE*	c.367-8C>A	Early onset IBD, oral aphtosis, recurrent gastrointestinal infections, severe congenital neutropenia
P83	Phagocyte defect	*CYBA*	c.70G>A p.Gly24Arg c.373G>A p.Ala125Thr	Colitis, perianal abscess, defective oxidative burst
P84	Autoinflammatory disorder	*ADA2*	c.319A>C p.Lys107Gln	EBV associated Hodgkin lymphoma, splenomegaly, anemia, hypogammaglobulinemia
P85	PIRD	*FAS*	c.761T>A p.Val254Asp	Lymphoproliferation, elevated DNT
P86	CID	*PNP*	c.461+1G>A	Autoimmune hemolytic anemia, neurological impairment, osteomyelitis, lymphopenia
P87	PIRD	*RAB27A*	c.514_518del p.Gln172AsnfsTer2	Preseptal cellulitis, partial albinism, cytopenia
P88	CID	*BACH2*	c.745del p.Ser249ValfsTer93	IBD, pancreatitis, hypogammaglobulinemia
P89	CID	*RNF31*	c.2846A>C p.Asn949Thr	Chronic diarrhea, hypoalbunemia, lymphoplasmacytic inflammation
P90	Autoinflammatory disorder	*ACP5*	c.772_790del Ser258Trpfs*39	Recurrent viral infections, thrombocytopenia, AIHA
P91	PIRD	*PRF1*	c.1122G>A p.Trp374Ter	Sepsis, HSM, cytopenia, recurrent moniliasis, HLH
P92	Phagocyte defect	*NCF1*	Exon 5-6 Dup	Necrotizing pneumonia, lymphopenia, neutropenia
P93	CID	*CHD7*	c.1904A>T p.Asp635Val	Facial dysmorphic features, recurrent pulmonary infections, chronic severe diarrhea, reduced CD3 lymphocytes
P94	CID	*FCHO1*	c.2183A>C p.Asn728Thr	BCG lymphadenitis, abdominal pain, hepatitis, elevated IgE, eosinophilia
P95	PIRD	*LRBA*	c.2836_2839del p.Glu946Ter	Recurrent pulmonary infections, IBD, panhypogammaglobulinemia, reduced switched memory B cells
P96	Innate immune defect	*TBK1*	c.1055T>C p.Leu352Pro	Enteroviral meningitis, recurrent sinopulmonary infections, failure to thrive
P97	SCID	*IL7R*	c.337G>T p.Glu113Ter	T- B+ NK+
P98	PIRD	*LRBA*	c.2836_2839del p.Glu946Ter	Recurrent sinopulmonary infections, CMV colitis, EBV, arthritis, deafness, hyper IgM phenotype, absent B lymphocytes
P99	SCID	*PRKDC*	c.9182T>G p.Leu3061Arg	T- B- NK+
P100	SCID	*RAG2*	c.104G>C p.Gly35Ala	T- B- NK+
P101	PAD/CVID	*PIK3CD*	c.1573G>A p.Glu525Lys	Lichen planus, fulminant hepatic failure, granuloma, ITP, lymphoproliferation, reduced switched memory B cells
P102	PIRD	*FAS*	c.340G>A p.Glu114Lys	AIHA, cytopenia, HSM, lymphoproliferation, crescentic GLN, agammaglobulinemia, elevated DNT, reduced Treg cells
P103	Innate immune defect	*STAT1*	c.1189A>G p.Asn3Asp	Recurrent pulmonary infections, bronchiectasis, CMC, nail dystrophia, severe growth retardation, hypothyroidism, hypergammaglobulinemia, CD4+ T cel lymphopenia
P104	CID	*IL6ST*	c.2093C>A p.Ala698Glu	Recurrent pulmonary infections, bronchiectasis, severe eczema, hypogammaglobulinemia, elevated IgE, lymphopenia
P105	Innate immune defect	*IL12RB1*	c.637C>T p.Arg213Trp	Severe pulmonary tuberculosis, vasculitis, recurrent arthritis
P106	CID	*DOCK8*	c.5766G>A p.Met1922Ile	Severe eczema, multiple food allergies, recurrent infections, elevated IgE, lymphopenia
P107	CID	*DOCK8*	Exon 1-10 Deletion	Recurrent infections, growth retardation, failure to thrive, food allergies, elevated IgE, hypogammaglobulinemia, lymphopenia
P108	CID	*SPINK5*	c.2658_2662dupGAGCA p.Ile888ArgfsTer56	Recurrent bacterial infections, failure to thrive, reduced memory B cells, elevated IgE,
P109	SCID	*ADA*	c.556G>A p.Glu186Lys	T- B- NK-
P110	CID	*RAG1*	c.2095C>T p.Arg699Trp	Erythroderma, severe recurrent infections, T cell lymphopenia
P111	PIRD	*PRF1*	c.1267delC p.Gln423LysfsX17	Sepsis, pancytopenia, HLH
P112	SCID	*IL2RG*	c.511G>T p.Glu171Ter	T- B+ NK-
P113	PAD/CVID	*CASP8*	c.919C>T p.Arg307Trp	Recurrent bacterial infections, HSM, hypogammaglobulinemia, low B cells, increased DNT cells
P114	CID	*DOCK8*	c.5831C>T p.Pro1944Leu	Recurrent pulmonary and cutaneous infections, bronchiectasis, T cell lymphopenia, high IgE
P115	SCID	*ADA*	c.556G>A p.Glu186Lys	T- B- NK-
P116	SCID	*RAG1*	c.1307C>A p.Thr436Asn	T- B- NK+
P117	SCID	*RAG1*	c.2322G>A p.Arg737His	T- B- NK+
P118	Phagocyte defect	*CYBA*	c.G70A p.Gly24Arg	Recurrent infections, lung granulomas, defective oxidative burst
P119	PIRD	*PRF1*	c.1385C>A p.Ser462Ter	Hemophagocytic lymphohistiocytosis HLH, HSM, low NK cells
P120	CID	*WAS*	c.37C>T p.Arg13Ter	Thrombocytopenia, eczema, recurrent bacterial infections, poor polysaccharide vaccine response
P121	CID	*WAS*	c.91G>A p.Glu31Lys	Thrombocytopenia, eczema, recurrent bacterial infections, low T cells
P122	PAD/CVID	*PIK3CD*	c.1573G>A p.Glu525Lys	EBV infection, lymphadenopathy, reduced IgA and IgG

*HSM* Hepatosplenomegaly, *ITP* Immune thrombocytopenic purpura, *IBD* Inflammatory bowel disease, *CMC* Chronic mucocutaneous candidiasis, *AIHA* Autoimmune hemolytic anemia, *SLE* Systemic lupus erythematosus, *HLH* Hemophagocytic lymphohistiocytosis, *RTE* recent thymic emigrant, *B-ALL* B-cell acute lymphoblastic leukemia, *MR* mental retardation, *DNT* Double negative T cells, *GLN* Glomerulonephritis

## Discussion

Advancements in NGS, with WES at the forefront, have been instrumental in the diagnostic processes of IEI by pinpointing causative genetic aberrations [72]. Genetic diagnosis now routinely assists in the delineation of IEI, underscoring its significance in the strategic management of patient treatments. Literature suggests a wide-ranging diagnostic yield for targeted and exome sequencing, from 10% to 70%, across various IEI patient groups [23, 58, 68, 73–79] . In this study, out of the 127 causative genetic defects in 122 patients, we identified 52 novel IEI-causing variants. We also discovered novel and very rare gene variants in *NFATC2, CHUK*, and *PIK3CG* genes, which have limited reported cases in the literature [80–83].

Among the 297 patients evaluated, a genetic etiology was confirmed in 122 individuals, resulting in a diagnostic yield of 41.1%. Diagnostic success exhibited pronounced variation among the different IEI subtypes: cases of SCID reached a 100% genetic identification rate, whereas CID and PID manifested lower diagnostic rates of 31% and 45%, respectively. Within the PAD cohort, genetic causality was determined in a mere 15.6% of cases (10 patients). This notably diminished diagnostic yield in Primary Antibody Deficiencies is in concordance with prior regional studies conducted by Fırtına S et al. [84]. In contrast, patients with probable Mendelian susceptibility to mycobacterial diseases and chronic granulomatous disease (CGD) demonstrated significantly higher diagnostic rates, with near-complete success in CGD patients.

The discrepancies in diagnostic success among IEI subtypes are primarily attributed to the complex nature of these disorders rather than limitations of WES. Factors such as the specific type of immunodeficiency, diverse clinical presentations, patient medical histories, and environmental influences affect the probability of achieving a genetic diagnosis [72]. Other factors include variable gene penetrance, the distinction between monogenic and polygenic influences, and various environmental considerations such as pathogenic exposures and age at presentation [85, 86]. Consanguinity plays a significant role in genetic diagnosis, as most IEI cases have autosomal recessive inheritance. Consanguineous populations or those from isolated regions with distinct phenotypes have reported higher diagnostic yields [87]. In our study, the consanguinity rate was 64.6%, and a diagnosis was made in 49.7% of those cases. We found 27 heterozygous variants in 21 unrelated patients, which can provide insights into the impact of heterozygous variants on protein function and aid in the search for novel IEI genes.

Currently, approximately 500 genetic etiologies leading to IEI are known [7]. Although the use of NGS, particularly WES, is increasing, it has limitations. Exome sequencing focuses on coding regions and essential splice sites, making it challenging to detect structural variations [72] and the use of short-read sequencing as in our study makes it difficult to map reads to repeated sequences, and pseudogenes [88]. Long-read sequencing (LRS) technologies both for exome or genome, have the capacity to enhance the detection of genetic variations and regions that are challenging to analyze with existing short-read NGS techniques [88–90]. However, the cost and complexity of analyzing large datasets pose challenges for WGS. In our study, we only identified three structural variants in 57 patients. Nevertheless, studies have shown the effectiveness of WGS in detecting both CNVs and coding variants [91, 92]. Reducing the cost of WGS and developing user-friendly bioinformatic tools may make it a routine diagnostic approach for IEI screening.

In conclusion, our findings highlight the limited success of WES in the genetic investigation of presumed IEI. The prospective adoption of WGS could enhance diagnostic yields, potentially surpassing WES in clinical examinations. With our substantial study cohort and diverse clinical presentations, the genetic variations we have identified will significantly contribute to the diagnosis of future IEI cases and guide the development of optimized NGS panels for these conditions.

## Supplementary Information

ESM 1 (PDF 806 kb)

## Data Availability

No datasets were generated or analysed during the current study.
