# Correction to: Genetic Evaluation of the Patients with Clinically Diagnosed Inborn Errors of Immunity by Whole Exome Sequencing: Results from a Specialized Research Center for Immunodeficiency in Türkiye

**DOI:** 10.1007/s10875-024-01841-3

**Published:** 2024-11-14

**Authors:** Baran Erman, Umran Aba, Canberk Ipsir, Damla Pehlivan, Caner Aytekin, Gokhan Cildir, Begum Cicek, Ceren Bozkurt, Sidem Tekeoglu, Melisa Kaya, Cigdem Aydogmus, Funda Cipe, Gulsan Sucak, Sevgi Bilgic Eltan, Ahmet Ozen, Safa Barıs, Elif Karakoc-Aydiner, Ayca Kıykım, Betul Karaatmaca, Hulya Kose, Dilara Fatma Kocacık Uygun, Fatih Celmeli, Tugba Arikoglu, Dilek Ozcan, Ozlem Keskin, Elif Arık, Elif Soyak Aytekin, Mahmut Cesur, Ercan Kucukosmanoglu, Mehmet Kılıc, Mutlu Yuksek, Zafer Bıcakcı, Saliha Esenboga, Deniz Çagdaş Ayvaz, Asena Pınar Sefer, Sukrü Nail Guner, Sevgi Keles, Ismail Reisli, Ugur Musabak, Nazlı Deveci Demirbas, Sule Haskologlu, Sara Sebnem Kilic, Ayse Metin, Figen Dogu, Aydan Ikinciogulları, Ilhan Tezcan

**Affiliations:** 1 Institute of Child Health, Hacettepe University, Ankara, Türkiye; 2 Can Sucak Research Laboratory for Translational Immunology, Hacettepe University, Ankara, Türkiye; 3 Department of Pediatric Immunology, Institute of Child Health, Hacettepe University, Ankara, Türkiye; 4 Pediatric Immunology, SBU Ankara Dr Sami Ulus Maternity Child Health and Diseases Training and Research Hospital, Ankara, Türkiye; 5 Centre for Cancer Biology, SA Pathology and the University of South Australia, Adelaide, SA 5000 Australia; 6 Department of Pediatric Allergy and Clinical Immunology, University of Health Sciences, Istanbul Basaksehir Cam and Sakura City Hospital, Istanbul, Türkiye; 7 Department of Pediatric Allergy and Clinical Immunology, Altinbas University School of Medicine, Istanbul, Türkiye; 8 Medical Park Bahçeşehir Hospital, Clinic of Hematology and Transplantation, İstanbul, Türkiye; 9 Marmara University, Faculty of Medicine, Department of Pediatric Allergy and Immunology, Jeffrey Modell Diagnostic and Research Center for Primary Immunodeficiencies, The Isil Berat Barlan Center for Translational Medicine, Istanbul, Türkiye; 10 Pediatric Allergy and Immunology, Cerrahpasa School of Medicine, Istanbul University-Cerrahpasa, Istanbul, Türkiye; 11 Department of Pediatric Allergy and Immunology, University of Health Sciences, Ankara Bilkent City Hospital, Ankara, Türkiye; 12 Department of Pediatric Immunology, Diyarbakir Children Hospital, Diyarbakır, Türkiye; 13 Division of Allergy Immunology, Department of Pediatrics, Akdeniz University Faculty of Medicine, Antalya, Türkiye; 14 Republic of Türkiye Ministry of Health Antalya Training and Research Hospital Pediatric Immunology and Allergy Diseases, Antalya, Türkiye; 15 Department of Pediatric Allergy and Immunology, Faculty of Medicine, Mersin University, Mersin, Türkiye; 16 Division of Pediatric Allergy and Immunology, Faculty of Medicine, Balcali Hospital, Cukurova University, Adana, Türkiye; 17 Department of Pediatric Allergy and Immunology, Faculty of Medicine, Gaziantep University, Gaziantep, Türkiye; 18 Department of Pediatric Allergy and Immunology, Etlik City Hospital, Ankara, Türkiye; 19 Division of Allergy and Immunology, Department of Pediatrics, Faculty of Medicine, University of Firat, Elazığ, Türkiye; 20 Department of Pediatric Immunology and Allergy, Faculty of Medicine, Zonguldak Bulent Ecevit University, Zonguldak, Türkiye; 21 Department of Pediatric Hematology, Faculty of Medicine, Ataturk University, Erzurum, Türkiye; 22 Department of Pediatrics, Division of Pediatric Immunology, Hacettepe University School of Medicine, Ankara, Türkiye; 23 Section of Pediatric Immunology, Institute of Child Health, Hacettepe University, Ankara, Türkiye; 24 Department of Pediatric Allergy and Immunology, Şanlıurfa Training and Research Hospital, Şanlıurfa, Türkiye; 25 Department of Pediatric Immunology and Allergy, Medicine Faculty, Necmettin Erbakan University, Konya, Türkiye; 26 Department of Immunology and Allergy, Baskent University School of Medicine, Ankara, Türkiye; 27 Department of Pediatric Immunology and Allergy, Ankara University Faculty of Medicine, Ankara, Türkiye; 28 Division of Pediatric Immunology-Rheumatology, Bursa Uludag University Faculty of Medicine, Bursa, Türkiye; 29 Translational Medicine, Bursa Uludag University, Translational Medicine, Bursa, Türkiye; 30 Department of Pediatrics, Division of Pediatric Immunology, Hacettepe University School of Medicine, Ankara, Türkiye

**Correction to: Journal of Clinical Immunology**

10.1007/s10875-024-01759-w

Since the publication of this article we have noticed several errors within the main Table 1 of the manuscript. Four variants were given with different transcript IDs of the same gene. There are also 2 nomenclature errors in the variants of P58 and P117. The necessary corrections have been made in the table below. The errors do not affect the causality of the variants, the results or conclusions reported in the manuscript.

The authors apologize for the error, and regret any inconvenience this may have caused.

The original version has been corrected.

**Table:** 

Patient no	Clinical diagnosis (IUIS)	Age	Gender	Consan	Gene	Variant	Transcript ID	Zygosity	Consequence	Novelty
P18	CID	20	F	+	*PGM3*	c.214G>A p.Gly72Ser	NM_001199917.1 The true RefseqID should be NM_001199919.1	Hom	Missense	Novel
P29	SCID	6 m	F	+	*ADA*	c.551_555del p.Glu184Glyfs*2 c.241G>A p.Gly81Arg	NM_000022.4 NM_000022.4 The true RefseqID should be NM_001322050 for these variants	Comp. Het	Out of frame/Deletion Missense	Novel rs2065384316
P34	SCID	1	M	+	*ADA*	c.779A>G p.Glu260Gly	NM_001322050 The true RefseqID should be NM_000022.4 for this variant	Hom	Missense	rs1354071013
P58	SCID	2	M	+	*DCLRE1C*	c.1633delT p.Glu545AsnfsTer The correct nomenclature of this variant is c.1633del p.Glu545Asnfs*58	NM_001350965.2	Hom	Out of frame/Deletion	Novel
P113	PAD/CVID	7	F	+	*CASP8*	c.919C>T p.Arg307Trp	NM_001372051.1 The true RefseqID should be NM_001080125.1	Hom	Missense	rs17860424
P117	SCID	1	F	+	*RAG1*	c.2322G>A p.Arg737His The correct nomenclature of this variant is c.2210G>A p.Arg737His The nucleotide position 2322 refers an old transcript	NM_000448.3	Hom	Missense	rs104894286

