# Supplementary material for: Genetic Evaluation of the Patients with Clinically Diagnosed Inborn Errors of Immunity by Whole Exome Sequencing: Results from a Specialized Research Center for Immunodeficiency in Türkiye
Source: J Clin Immunol. 2024 Jul 2;44(7):157. doi: 10.1007/s10875-024-01759-w (PMC11219406; doi:10.1007/s10875-024-01759-w)
Supplement: Supplementary file 1 — (PDF 806 kb) [file 10875_2024_1759_MOESM1_ESM.pdf]

Genetic evaluation of the patients with clinically diagnosed inborn errors of immunity by whole exome sequencing: Results from a specialized research center for immunodeficiency in Türkiye

Journal of Clinical Immunology

Baran Erman[1,2], Umran Aba[2,3], Canberk Ipsir[2,3], Damla Pehlivan[2], Caner Aytekin[4], Gökhan Cildir[5], Begum Cicek[1], Ceren Bozkurt[2], Sidem Tekeoğlu[2], Melisa Kaya[2], Ciğdem Aydoğmus[6], Funda Cipe[7], Gülsan Sucak[8], Sevgi Bilgic Eltan[9], Ahmet Özen[9], Safa Barış[9], Elif Karakoç-Aydiner[9], Ayca Kıykım[10], Betul Karaatmaca[11], Hulya Kose[12], Dilara Fatma Kocacık Uygun[13], Fatih Çelmeli[14], Tuğba Arıkoğlu[15], Dilek Özcan[16], Özlem Keskin[17], Elif Arık[17], Elif Soyak Aytekin[18], Mahmut Cesur[17], Ercan Küçükosmanoğlu[17], Mehmet Kılıç[19], Mutlu Yüksek[20], Zafer Bıçakçı[21], Saliha Esenboğa[22], Deniz Çağdaş Ayvaz[22,23], Asena Pınar Sefer[24], Sükrü Nail Güner[25], Sevgi Keleş[25], İsmail Reisli[25], Uğur Muşabak[26], Nazlı Deveci Demirbas[27], Şule Haskoloğlu[27], Sara Sebnem Kılıc[28,29], Ayşe Metin[11], Figen Doğu[27], Aydan İkincioğulları[27*], İlhan Tezcan[30*]

[1] Institute of Child Health, Hacettepe University, Ankara, Türkiye

[2] Can Sucak Research Laboratory for Translational Immunology, Hacettepe University, Ankara, Türkiye

[3] Department of Pediatric Immunology, Institute of Child Health, Hacettepe University, Ankara, Türkiye

[4] Pediatric Immunology, SBU Ankara Dr Sami Ulus Maternity Child Health and Diseases Training and Research Hospital, Ankara, Türkiye

[5] Centre for Cancer Biology, SA Pathology and the University of South Australia, Adelaide, SA 5000, Australia

[6] Department of Pediatric Allergy and Clinical Immunology, University of Health Sciences, Istanbul Basaksehir Cam and Sakura City Hospital, Istanbul, Türkiye

[7] Department of Pediatric Allergy and Clinical Immunology, Altinbas University School of Medicine, Istanbul, Türkiye

[8] Medical Park Bahçeşehir Hospital, Clinic of Hematology and Transplantation, İstanbul, Türkiye

[9] Marmara University, Faculty of Medicine, Department of Pediatric Allergy and Immunology, Jeffrey Modell Diagnostic and Research Center for Primary Immunodeficiencies, The Isil Berat Barlan Center for Translational Medicine, Istanbul, Türkiye

[10] Pediatric Allergy and Immunology, Cerrahpasa School of Medicine, Istanbul University-Cerrahpasa, Istanbul, Türkiye

[11] Department of Pediatric Allergy and Immunology, University of Health Sciences, Ankara Bilkent City Hospital, Ankara, Türkiye

[12] Department of Pediatric Immunology, Diyarbakir Children Hospital, Diyarbakır, Türkiye

[13] Division of Allergy Immunology, Department of Pediatrics, Akdeniz University Faculty of Medicine, Antalya, Türkiye

[14] Republic of Türkiye Ministry of Health Antalya Training and Research Hospital Pediatric Immunology and Allergy Diseases, Antalya, Türkiye

[15] Department of Pediatric Allergy and Immunology, Faculty of Medicine, Mersin University, Mersin, Türkiye

[16] Division of Pediatric Allergy and Immunology, Faculty of Medicine, Balcali Hospital, Cukurova University, Adana, Türkiye

[17] Department of Pediatric Allergy and Immunology, Faculty of Medicine, Gaziantep University, Gaziantep, Türkiye

[18]Department of Pediatric Allergy and Immunology, Etlik City Hospital, Ankara, Türkiye

[19] Division of Allergy and Immunology, Department of Pediatrics, Faculty of Medicine, University of Firat, Elazığ, Türkiye

[20] Department of Pediatric Immunology and Allergy, Faculty of Medicine, Zonguldak Bulent Ecevit University, Zonguldak, Türkiye

[21] Department of Pediatric Hematology, Faculty of Medicine, Ataturk University, Türkiye

[22] Hacettepe University School of Medicine, Department of Pediatrics, Division of Pediatric Immunology, Ankara, Türkiye

[23] Section of Pediatric Immunology, Institute of Child Health, Hacettepe University, Ankara, Türkiye

[24]Department of Pediatric Allergy and Immunology, Şanlıurfa Training and Research Hospital, Şanlıurfa, Türkiye

[25] Department of Pediatric Immunology and Allergy, Medicine Faculty, Necmettin Erbakan University, Konya, Türkiye

[26] Department of Immunology and Allergy, Baskent University School of Medicine, Ankara, Ankara, Türkiye

[27] Department of Pediatric Immunology and Allergy, Ankara University Faculty of Medicine, Ankara, Türkiye

[28] Division of Pediatric Immunology-Rheumatology, Bursa Uludag University Faculty of Medicine, Bursa, Türkiye

[29] Translational Medicine, Bursa Uludag University, Translational Medicine, Bursa, Türkiye

[30] Hacettepe University School of Medicine, Department of Pediatrics, Division of Pediatric Immunology, Ankara, Türkiye

*These authors contributed equally to this work

Correspondence: Baran Erman, baranerman@gmail.com

Keywords: Inborn errors of immunity, next generation sequencing, whole exome sequencing, genetic diagnosis

Supplementary Tables:

Supplementary Table 1. Pathogenicity of the variants

Patient no	Gene	Variant	ACMG-based pathogenity	Evidence Code	CADD	Polyphen	Gnomad*	Turkish variome [1]#
**P1**	*CARD9*	c.883C>T p.Gln295Ter	P	PVS1 PP5	41	-	0	0.0001783
**P2**	*RFXANK*	c.634C>T p.Arg212Ter	P	PP5 PVS1 PM2	39	-	0	0.0003643
**P3**	*CD3E*	c.176G>A p.Trp59Ter	P	PP5 PVS1 PM2	35	-	0	
**P4**	*NFATC2*	c.340_345delGAGATC p.Glu114_Ile115del	VUS	PM2	21	-	0	
**P5**	*JAK3*	c.2134G>A p.Gly712Ser	VUS	PM2	25.7	PD	0	
**P6**	*RAG2*	c.581C>A p.Ser194Ter	P/LP	PP5 PVS1 PM2	36	-	0	
**P7**	*RAG1*	c.2005G>Ap.Glu669Lys<br>c.1307C>A p.Thr436Asn	P/LP<br>VUS	PP5 PM2<br>PM2 BP4	28.2<br>23.8	D<br>D	0<br>0	
**P8**	*RAG1*	c.2005G>Ap.Glu669Lys<br>c.1307C>A p.Thr436Asn	VUS<br>VUS	PM2 BP4<br>PM2 BP4	24.1<br>28.2	D<br>D	0<br>0	
**P9**	*CD70*	c.332C>T p.Thr111Met	P/LP	PP5 PM2	25.3	D	0	
**P10**	*CD70*	c.332C>T p.Thr111Met	P/LP	PP5 PM2	25.3	D	0	
**P11**	*CYBA*	c.58+4_58+7delAGTG	LP	PP5	-	-	0	
**P12**	*ZNF341*	c.1626C>G p.Tyr542Ter	P	PP5 PVS1 PM2	36	-	0	
**P13**	*ZAP70*	c.1010T>G p.Leu337Ala	P	PP5 PM2	28.4	D	0	
**P14**	*RAG2*	c.105G>C p.Gly35Ala	P	PP5 PM2	24.4	D	0	
**P15**	*RAG2*	c.105G>C p.Gly35Ala	P	PP5 PM2	24.4	D	0	
**P16**	*TNFRSF13B*	c.310C>T p.Cys104Arg	LP	PP5 PM2	25.8	D	0	0.003123141
**P17**	*PIK3R1*	c.837-1G>A	VUS	PM2	-	-	0	
**P18**	*PGM3*	c.214G>A p.Gly72Ser	LP	PP5 PM2	29.9	D	0	
**P19**	*SAMD9L*	c.2639A>C p.His880Pro	VUS	PM2	24.2	B	0	
**P20 5**	*TNFRSF13B*	c.204dupA p.Leu69Tfs*11	P	PP5 PVS1	22.8	-	1	
**P21**	*CD79A*	c.380-2A>G	VUS		-	-	0	
**P22**	*DNMT3B*	c.2029G>A p.Val677Met	LP	PP5 PM2	27.2	D	0	
**P23**	*AICDA*	c.A100T p.Lys34Ter	P	PVS1 PP5	37	-	0	
**P24**	*CYBA*	c.G70A p.Gly24Arg	P/LP	PP5 PM2	26.4	D	0	
**P25**	*MALT1*	c.1318_1321delTGTC p.L440Valfs*6	VUS	PM2	-	-	0	
**P26**	*SBDS*	c.578T>C p.Lys193Pro	VUS	PM2	45	PD	0	

		c.184A>T p.Lys62Ter	VUS	PM2	28.1	PD	0	
**P27**	*RFXANK*	Exon 2-6 Deletion	VUS	PM2	-	-	0	
**P28**	*MAGT1*	c.199-16A>G	VUS	PM2	-	-	0	
**P29**	*ADA*	c.551_555del p.Glu184Glyfs*2	LP	PVS1 PM2	-	-	0	
		c.241G>A p.Gly81Arg	VUS	PM2	22.1	-	0	
**P30**	*RAG1*	c.1767C>G p.Tyr589Ter	LP	PVS1 PM2	32	-	0	0.000297619
**P31**	*JAK3*	c.932delC p.Pro311Argfs*17	LP	PVS1 PM2	-	-	0	
**P32**	*TRAF3IP2*	c.559C>T p.Arg187Ter	LP	PVS1 PM2	36	-	0	
**P33**	*RAG1*	c.2126G>A p.Gly709Asp	VUS	PM2	23.9	D	0	
**P34**	*ADA*	c.779A>G p.Glu260Gly	LP	PM2 PP3	28.5	D	-	
**P35**	*NCF2*	c.233G>A p.Gly78Glu	LP	PM2 PP3	25.7	D	0	
**P36**	*CYBA*	c.166dupC p.Arg56Profs*156	LP	PVS1	-	-	0	
**P37**	*LRBA*	c.646-1G>A	VUS	PM2	-	-	0	
**P38**	*JAK3*	c.2080G>T p.Glu694Ter	LP	PVS1 PM2	32	-	0	
**P39**	*IL2RG*	c.437T>A p.Leu146Gln	VUS	PM2 PP3	23.3	PD	0	
**P40**	*PRKCD*	c.1097G>A p.Gly366Glu	VUS	PM2 BP4	22.9	PD	0	
**P41**	*RAG2*	c.623T>A p.Val208Asp	VUS	PM3 BP3	27	PD	0	
**P42**	*CTLA4*	c.118G>A p.Val40Met	LP	PM2 BP4 PP5	24.3	PD	0	
**P43**	*JAK1*	c.2485A>G p.Asn829Asp	VUS	PM2 BP4	23.7	PD	0	
**P44**	*RAG1*	c.1767C>G p.Tyr589Ter	LP	PVS1 PM2	32	-	0	0.000297619
**P45**	*PRKCD*	c.1097G>A p.Gly366Glu	VUS	PM2 BP4	22.9	PD	0	
**P46**	*CYBB*	c.770G>A p.Cys257Tyr	VUS	PM2 PP3	23.5	PD		
**P47**	*CHUK*	c.499G>A p.Gly167Arg	VUS	PM2 PP3	28	PD	0	
**P48**	*CHUK*	c.499G>A p.Gly167Arg	VUS	PM2 PP3	28	PD	0	
**P49**	*RAG1*	c.742C>T p.Gln248Ter	LP	PVS1 PM2	32	-	0	
**P50**	*CD40L*	c.15C>A p.Tyr5Ter	P	PP5 PVS1 PM2	32	-	0	
**P51**	*UNC13D*	c.2346_2349delGGAG p.Arg782SerfsTer12	P	PP5 PVS1 PM2	-	-	0	0.000646831
**P52**	*IGGL1*	c.425C>T p.Pro142Leu	VUS	PM2 BP4	15.4	PD	0	
**P53**	*ELANE*	c.703delG p.Val235TrpfsTer5	VUS	PM2	-	-	0	

**P54**	*HCK*	c.135_136delinsTG p.Pro46Ala	VUS	PM2	-	-	0	
**P55**	*CYBA*	c.385G>A p.Glu129Lys	VUS	PM2 PP3	26.1	PD	0	
**P56**	*SLC7A7*	c.1417C>T p.Arg473Ter	VUS	PM2	31	-	0	
**P57**	*NCF2*	c.196C>T p.Arg66Ter	P	PP5 PVS1 PM2	54	-	0	
**P58**	*DCLRE1C*	c.1633delT p.Glu545AsnfsTer	LP	PVS1 PM2	-	-	0	
**P59**	*RAG2*	c.712delC p.Val238LeufsTer10	LP	PVS1 PM2	-	-	0	0.0003541
**P60**	*IL12RB1*	c.523C>T p.Arg175Trp	VUS	PM2 PP3	22.5	PD	0	
**P61**	*CD40L*	c.15C>A p.Tyr5Ter	P	PP5 PVS1 PM2	32	-	0	
**P62**	*ADA2*	c.1072G>A p.Gly358Arg	VUS	PM2 PP3	25.2	PD	0	
**P63**	*IL12RB1*	c.1456C>T p.Arg486Ter	P	PP5 PVS1 PM2	33	-	0	
**P64**	*CHUK*	c.499G>A p.Gly167Arg	P	PP5 PVS1 PM2	32	-	0	
**P65**	*CYBA*	c.371C>T p.Ala124Val	VUS	PM2 PP3	24.1	PD	0	
**P66**	*GIMAP5*	c.667C>T p.Leu223Phe	P/LP	PP5 PM2	22.7	PD	0	
**P67**	*GIMAP5*	c.667C>T p.Leu223Phe	P/LP	PP5 PM2	22.7	PD	0	
**P68**	*CD79A*	c.177dup p.Asn60GlnfsTer20	LP	PVS1 PM2	-	-	0	
**P69**	*UNC13D*	c.1082del p.Tyr361SerfsTer43	LP	PVS1 PM2	-	-	0	
**P70**	*FAS*	c.361C>T p.Arg121Trp	P/LP	PP5 PM2	22.2	D	0	
**P71**	*PRF1*	c.1122G>A p.Trp374Ter	LP	PP5 PM2	47	-	0	0.001022495
**P72**	*DOCK8*	c.5831C>T p.Pro1944Leu	VUS	PM2 PP3	32	PD	0	
**P73**	*DOCK8*	c.5831C>T p.Pro1944Leu	VUS	PM2 PP3	32	PD	0	
**P74**	*CTLA4*	c.151C>T p.Arg51Ter	P/LP	PP5 PVS1 PM2	34	-	0	
**P75**	*HAX1*	c.130_131insA p.Trp44Ter	P	PP5 PVS1 PM2	-	-	0	0.000646831
**P76**	*PIK3CG*	c.2159A>G p.Tyr720Cys	VUS	PM2 PP3	29.6	PD	0	
**P77**	*MALT1*	c.1133T>G p.Phe378Cys	VUS	PM2 PP3 BP1	28.7	PD	0	
**P78**	*MAGT1*	c.628-4T>C	VUS	PM2	-	-	0	
**P79**	*ACP5*	c.772_790del p.Ser258WTrpfs*39	VUS	PP5 PM2	-	-	0	
**P80**	*PGM3*	c.821A>G p.Asn274Ser	LP	PP5 PM2 PP3	24.4	PD	0	
**P81**	*CD3G*	c.80-1G>C	VUS	PM2	-	-	0	

**P82**	*ELANE*	c.367-8C>A	VUS	PP5 PM2	-	-	0	
**P83**	*CYBA*	c.70G>Ap.Gly24Arg<br>c.373G>A p.Ala125Thr	P<br>P	PP5 PM2 PP3<br>PP5 PM2	25<br>24.6	PD<br>D	0<br>0	
**P84**	*ADA2*	c.319A>C p.Lys107Gln	VUS	PM2 PM3	24	PD	0	
**P85**	*FAS*	c.761T>A p.Val254Asp	VUS	PM1 PM2 PP3	23.5	PD	0	
**P86**	*PNP*	c.461+1G>A	LP	PVS1 PM2	-	-	0	
**P87**	*RAB27A*	c.514_518del p.Gln172AsnfsTer2	LP	PP5 PVS1 PM2	-	-	0	
**P88**	*BACH2*	c.745del p.Ser249ValfsTer93	LP	PVS1 PM2	-	-	0	
**P89**	*RNF31*	c.2846A>C p.Asn949Thr	VUS	PM2 BP4	24.1	PD	0	
**P90**	*ACP5*	c.772_790del Ser258Trpfs*39	LP	PP5 PVS1 PM2	-	-	0	
**P91**	*PRF1*	c.1122G>A p.Trp374Ter	LP	PP5 PVS1 PM2	47	-	0	0.001022495
**P92**	*NCF1*	Exon 5-6 Dup	VUS		-	-	0	
**P93**	*CHD7*	c.1904A>T p.Asp635Val	VUS	PM2 BP4	27	PD	0	
**P94**	*FCHO1*	c.2183A>C p.Asn728Thr	VUS	PM2 BP4	23.1	PD	0	
**P95**	*LRBA*	c.2836_2839del p.Glu946Ter	P	PP5 PVS1 PM2	-	-	0	
**P96**	*TBK1*	c.1055T>C p.Leu352Pro	VUS	PM2 BP4	26.2	PD	0	
**P97**	*IL7R*	c.337G>T p.Glu113Ter	LP	PVS1 PM2	25.8	-	0	
**P98**	*LRBA*	c.2836_2839del p.Glu946Ter	P	PM5 PVS1 PM2	-	-	0	
**P99**	*PRKDC*	c.9182T>G p.Leu3061Arg	VUS	PP5 PM2 BP4	26.4	PD	0	
**P100**	*RAG2*	c.104G>C p.Gly35Ala	LP	PM2 PM5 PP2	25.3	D	0	
**P101**	*PIK3CD*	c.1573G>A p.Glu525Lys	P/LP	PP5 PM2	24.8	PD	0	
**P102**	*FAS*	c.340G>A p.Glu114Lys	VUS	PM2 PP3	25.8	PD	0	
**P103**	*STAT1*	c.1192G>A p.Gly397Ser	VUS	PM2 PP3	32	PD	0	
**P104**	*IL6ST*	c.2093C>A p.Ala698Glu	VUS	PM2 BP4	23.2	PD	0	
**P105**	*IL12RB1*	c.637C>T p.Arg213Trp	P	PP5 PM2	19.1	D	0	
**P106**	*DOCK8*	c.5766G>A p.Met1922Ile	VUS	PM2 BP4	22.6	PD	0	
**P107**	*DOCK8*	Exon 1-10 Deletion	VUS	PM2	-	-	0	

**P108**	*SPINK5*	c.2658_2662dupGAGCA p.Ile888ArgfsTer56	VUS	PM2	-	-	0	
**P109**	*ADA*	c.556G>A p.Glu186Lys	LP	PM2 BP4	26	D	0	
**P110**	*RAG1*	c.2095C>T p.Arg699Trp	P/LP	PP5 PM2	26	D	0	
**P111**	*PRF1*	c.1267delC p.Gln423LysfsX17	LP	PVS1 PM2	-	-	0	
**P112**	*IL2RG*	c.511G>T p.Glu171Ter	P	PM5 PVS1 PM2	24.3	-	0	
**P113**	*CASP8*	c.919C>T p.Arg307Trp	LP	PM2 PM5 PP2	23.4	D	0	
**P114**	*DOCK8*	c.5831C>T p.Pro1944Leu	LP	PM2 PM5 PP2	27.1	D	0	
**P115**	*ADA*	c.556G>A p.Glu186Lys	LP	PM2 BP4	26	D	0	
**P116**	*RAG1*	c.1307C>A p.Thr436Asn	VUS	PM2 BP4	24.1	D	0	
**P117**	*RAG1*	c.2322G>A p.Arg737His	P/LP	PP5 PM2	29.6	D	0	0.000177
**P118**	*CYBA*	c.70G>A p.Gly24Arg	VUS	PP5 PM2 PP3	25	PD	0	
**P119**	*PRF1*	c.1385C>A p.Ser462Ter	P	PM5 PVS1 PM2	26.6	-	0	
**P120**	*WAS*	c.37C>T p.Arg13Ter	P	PM5 PVS1 PM2	34	-	0	
**P121**	*WAS*	c.91G>A p.Glu31Lys	P/LP	PP5 PM2	26	PD	0	
**P122**	*PIK3CD*	c.1573G>A p.Glu525Lys	P/LP	PP5 PM2	24.8	PD	0	

CADD: Combined Annotation Dependent Depletion ACMG: American College of Medical Genetics and Genomics VUS: Variant of Uncertain Significance *Number of homozygous individuals in Gnomad 4.1 #Allele frequencies in Turkish Variome

ACMG-based pathogenicity: Our data analysis application "Seq, Genomize" uses ACMG 2015 criteria with minor modifications as explained below:

Evidence Codes (Autopathogenicity v2.x)

PVS1

A Null variant (nonsense, frameshift, canonical ±1 or 2 splice sites, initiation codon, single or multiexon deletion) in a gene where the LOF is a known mechanism of a disease.

Null variant types:

Stop gained/Frameshift (Nonsense or Frameshift variant)

Splice acceptor/donor (GT-AG, 1,2 splice sites)

Start lost (Initiation Codon)

Deletion (Inframe or full gene deletion)

Loss of function is a mechanism of the disease if (either of the below is required):

At least 3 pathogenic LoF variants from the ClinVar (with a minimum of 1 star) need to be present

The LOEUF score of the transcript is smaller than 0.35 as suggested by GNOMAD

The transcript is biologically relevant if it is present in:

All RefSeq transcripts

Ensembl transcripts with expression in any of the 51 GTeX (v6) tissues (excluding expression data from two cell types)

The LoF variant in a particular exon is frequent in the general population if:

The 99th percentile of the allele frequency distribution of the previously reported LoF variants (GnomAD) in the exon is higher than the predefined threshold of 0.1%.

The Truncated/Altered region is critical for the protein function if:

For the nonsense/frameshift mutations: The altered region is the region between the variant position and the last base of the coding sequence (excluding the stop codon).

For the splice mutations: If the ORF is preserved, we consider the nearby (upstream for splice donor, downstream for splice acceptor) skipped exon as the altered region. Otherwise, it is the region between the variant position and the last base of the coding sequence.

For the deletion mutations: If the ORF is preserved, we consider only the skipped region of the protein. Otherwise, it is the region between the variant position and the last base of the coding sequence.

For the altered region to be critical for the protein, we require at least 3 pathogenic ClinVar variants with a minimum of 2 stars (multiple submitters) or higher.

Expected to undergo NMD (nonsense-mediated decay) if:

The variant is not present in the 3' most coding exon or the 3' most 50 bp of the penultimate coding exon.

The transcript has a multi-coding exon.

PS1

The same amino acid change as a previously established pathogenic variant regardless of the nucleotide change.

Evidence code strength is modified based on the Clinvar review status:

1-star: PS1 (Supporting)

2-star: PS1 (Moderate)

3-4 star: PS1 (Strong)

PM1

Located in a mutational hotspot and/or critical and well-established functional domain (e.g., an active site of an enzyme) without benign variation.

Detection of a mutational hotspot

A hotspot region is defined as the largest pathogenic variant dense region between two benign variations and meeting the following conditions:

Presence of a minimum of 5 pathogenic and 0 benign variants reported in the ClinVar.

Pathogenic variant count per base pair of the region is greater than the predefined threshold of 0.2.

Functional UniProt domain

To define a functional UniProt domain, the following rules are employed:

UniProt domains with ('chain', 'coiled-coil region', 'transit peptide', 'helix', 'turn', 'beta strand') categories are excluded.

Domains without benign variation in the ClinVar (with a minimum of 1 star).

At least 3 pathogenic variants in the ClinVar (with a minimum of 1 star).

PM2

Absent from controls (or at extremely low frequency if recessive) in the Exome Sequencing Project, the 1000 Genomes Project, or the Exome Aggregation Consortium databases.

Maximum allele frequency of the variant is less than the predefined threshold.

PM4

The protein length changes as a result of in-frame deletions/insertions in a non-repeat region or stop-loss variants.

PM5

A novel missense change at an amino acid residue where a different missense change was previously observed as a pathogenic variant.

Evidence code strength modified based on the Clinvar review status:

1-2 star: PM5 (Supporting)

3-4 star: PM5 (Moderate)

PP2

A missense variant in a gene that has a low rate of benign missense variation and in which missense variants are a common mechanism of disease.

This rule only applies to missense variants. The following thresholds are applied to determine if a missense variant is a common mechanism of disease:

Minimum missense pathogenic variant count in the transcript: 5

Missense/total pathogenic variant count ratio threshold: 70%

Missense pathogenic/benign count threshold: 0.65

PP3

Multiple lines of computational evidence support a deleterious effect on the gene or the gene product (conservation, evolutionary, splicing impact, etc.).

PP3 is assigned if one the following criteria is met (Ionnadis et al. 2016 (opens new window)):

The REVEL score is greater than 0.75

The REVEL score is greater than 0.5 and MetalR is damaging

The REVEL score is not available and MetalR is damaging

PP5

A reputable source recently reported the variant as pathogenic, but the evidence is not available to the laboratory to perform an independent evaluation.

Evidence code strength is modified based on the Clinvar review status:

1-star: PP5 (Supporting)

2-star: PP5 (Moderate)

3-star: PP5 (Strong)

4-star: PP5 (Very strong)

BA1

The allele frequency is >5% in the Exome Sequencing Project, the 1000 Genomes Project, or the Exome Aggregation Consortium databases.

Maximum allele frequency of the variant is greater than 5%

Variants on the BA1 exception list provided by Clingen are excluded. Clingen Exception List(opens new window)

BS1

The allele frequency is greater than the expected frequency for the disorder.

Maximum allele frequency of the variant is less than the predefined BS1 threshold

BS2

Observed in a healthy adult individual for a recessive (homozygous), dominant (heterozygous), or X-linked (hemizygous) disorder, with full penetrance expected at an early age.

A variant was observed (at least 2 times) in the control cohort of the Gnomad (v2.1.1) database in a homozygous state.

BP1

A Missense variant in a gene for which primarily truncating variants are known to cause disease.

At least 5 pathogenic variants are reported in the ClinVar database.

All pathogenic variants in the ClinVar database are truncating.

BP3

In-frame deletions/insertions in a repetitive region without a known function.

BP3 is assigned if the variant is in a repetitive region and the repetitive region meets the following criteria.

Do not overlap with a functional UniProt domain.

Do not have a pathogenic variant reported in the ClinVar.

BP4

Multiple lines of computational evidence suggest no impact on the gene or the gene product (conservation, evolutionary, splicing impact, etc.).

BP4 is assigned if one the following criteria is met (Ionnadis et al. 2016 (opens new window)):

The REVEL score is less than 0.5

The REVEL score is less than 0.75 and MetalR is benign

The REVEL score is not available and MetalR is benign

BP6

A Reputable source recently reported the variant as benign, but the evidence is not available to the laboratory to perform an independent evaluation.

Evidence code strength is modified based on ClinVar review status:

1-2-star: BP6 (Supporting)

3-star: BP6 (Strong)

4-star: BP6 (Stand alone)

BP7

A synonymous (silent) variant for which splicing prediction algorithms predict no impact to the splice consensus sequence nor the creation of a new splice site AND the nucleotide is not highly conserved.

BP7 is assigned if the following criteria are met:

Cryptic splice site is not predicted (ADA and RF scores are less than 0.6)

Low conservation score (phylop100way score is less than 1.879)

Unimplemented Evidence Codes

The following evidence codes are unimplemented: PS2, PS3, PS4, PM3, PM6, PP1, PP4, BS3, BS4, BP2, BP5.

Supplementary Table 2: Detailed evaluation of possible disease-causing monoallelic variants

Patient no	Gene	Variant	CADD	ACMG criteria				
**P42**	*CTLA4*	c.118G>A p.Val40Met	24.3	LP	AIHA, enteropathy, reduced T and B cells	rs155365737 8	[2-5]	-
**P53**	*ELANE*	c.703delG p.Val235TrpfsTer5	-	VUS	Recurrent bacterial infections, severe congenital neutropenia	Novel	-	Congenital neutropenia
**P70**	*FAS*	c.361C>T p.Arg121Trp	22.1	Pathogenic/ Likely Pathogenic	Splenomegaly, lymphadenopathy, ITP	rs121913078	[6]	-
**P82**	*ELANE*	c.367-8C>A	-	VUS	Early onset IBD, oral aphtosis, recurrent gastrointestinal infections, severe congenital neutropenia	novel	-	Reduced/absence expression of ELANE by western blot
**P88**	*BACH2*	c.745del p.Ser249ValfsTer93	-	Likely Pathogenic	IBD, pancreatitis, hypogammaglobulinemia	novel	-	Reduced/absence expression of BACH2 by western blot
**P96**	*TBK1*	c.1055T>C p.Leu352Pro	26.1	VUS	Enteroviral meningitis, recurrent sinopulmonary infections, failure to thrive	novel	-	Impaired immune response to TLR3-dependent viruses

					lymphoproliferation, reduced switched memory B cells			
**P102**	*FAS*	c.340G>A p.Glu114Lys	26.5	VUS	Missense	rs773565107	-	Aberrant lymphocyte apoptosis
**P103**	*STAT1*	c.1192G>A p.Gly397Ser	28.9	VUS	Recurrent pulmonary infections, bronchiectasis, CMC, nail dystrophia, severe growth retardation, hypothyroidism, hypergammaglobulinemia, CD4+ T cel lymphopenia	novel	-	
**P104**	*IL6ST*	c.2093C>A p.Ala698Glu	23.3	VUS	Recurrent pulmonary infections, bronchiectasis, severe eczema, hypogammaglobulinemia, elevated IgE, lymphopenia	rs745818447	-	
**P122**	*PIK3CD*	c.1573G>A p.Glu525Lys	29.6	Pathogenic	EBV infection, lymphadenopathy, reduced IgA and IgG	rs587777389	[9, 10]	-

CADD: Combined Annotation Dependent Depletion ACMG: American College of Medical Genetics and Genomics VUS: Variant of Uncertain Significance
AIHA: Autoimmune hemolytic anemia HSM: Hepatosplenomegaly IBD: Inflammatory bowel disease ITP: Immune thrombocytopenic purpura DNT: Double negative T cells

Supplementary Table 3. Variants detected in all kindreds in the study

Patients and kindreds	Age	Gender	Gene	Variant	Characteristics
**K1 (P7-P8)**	2 - 1	M - M	RAG1	c.2005G>A p.Glu669Lys c.1307C>A p.Thr436Asn	P7: T- B- NK+ SCID, very low T and B cells<br><br>P8: T- B- NK+ SCID, very low T and B cells
**K2 (P9-P10)**	6- 4	M- M	CD70	c.332C>T p.Thr111Met	P9: Burkitt lymphoma, chronic diarrhea, recurrent pneumonia, EBV LAP, hypogammaglobulinemia, reduced memory B cells<br><br>P10: Non-Hodgkin lymphoma, recurrent pneumonia, EBV LAP, hypogammaglobulinemia, reduced memory B cells
**K3 (P14-P15)**	1 - 1	M - M	RAG2	c.105G>C p.Gly35Ala	P14: T- B- NK+ SCID, very low T and B cells<br><br>P15: T- B- NK+ SCID, very low T and B cells
**K4 (P47-P48)**	8 - 4	F - F	CHUK	c.499G>A p.Gly167Arg	P47: Recurrent bacterial, viral, fungal infections, chronic diarrhea, failure to thrive, hepatic fibrosis, absent secondary lymphoid tissues, hypogammaglobulinemia, reduced switched memory B cells<br><br>P48: Recurrent bacterial, viral, fungal infections, chronic diarrhea, failure to thrive, hepatic fibrosis, absent secondary lymphoid tissues, hypogammaglobulinemia, reduced switched memory B cells
**K5 (P66-P67)**	17 - 12	M - F	GIMAP5	c.667C>T p.Leu223Phe	P66: Hodgkin lymphoma, food allergy, family history, reduced IgG, low B cells, slightly reduced T cell activation<br><br>P67: Hodgkin lymphoma, family history, reduced IgG, low B cells, slightly reduced T cell activation
**K6 (P72-P73)**	4 - 6	M - F	DOCK8	c.5831C>T p.Pro1944Leu	P72: Failure to thrive, HPV infection, verrucous lesion, EBV +, CD4 T cell lymphopenia, elevated IgE<br><br>P73: Failure to thrive, HPV infection, verrucous lesion, CD4 T cell lymphopenia, elevated IgE

K: Kindred SCID: Severe combined immunodeficiency LAP: Lymphadenopathy

References

1. Kars ME, Basak AN, Onat OE, Bilguvar K, Choi J, Itan Y, et al. The genetic structure of the Turkish population reveals high levels of variation and admixture. Proc Natl Acad Sci U S A. 2021;118(36).
2. Egg D, Rump IC, Mitsuiki N, Rojas-Restrepo J, Maccari ME, Schwab C, et al. Therapeutic options for CTLA-4 insufficiency. J Allergy Clin Immunol. 2022;149(2):736-46.
3. Hoshino A, Tanita K, Kanda K, Imadome KI, Shikama Y, Yasumi T, et al. High frequencies of asymptomatic Epstein-Barr virus viremia in affected and unaffected individuals with CTLA4 mutations. Clin Immunol. 2018;195:45-8.
4. Rae W, Ward D, Mattocks C, Pengelly RJ, Eren E, Patel SV, et al. Clinical efficacy of a next-generation sequencing gene panel for primary immunodeficiency diagnostics. Clin Genet. 2018;93(3):647-55.
5. Schwab C, Gabrysch A, Olbrich P, Patino V, Warnatz K, Wolff D, et al. Phenotype, penetrance, and treatment of 133 cytotoxic T-lymphocyte antigen 4-insufficient subjects. J Allergy Clin Immunol. 2018;142(6):1932-46.
6. Bettinardi A, Brugnoni D, Quiros-Roldan E, Malagoli A, La Grutta S, Correra A, Notarangelo LD. Missense mutations in the Fas gene resulting in autoimmune lymphoproliferative syndrome: a molecular and immunological analysis. Blood. 1997;89(3):902-9.
7. Kuehn HS, Ouyang W, Lo B, Deenick EK, Niemela JE, Avery DT, et al. Immune dysregulation in human subjects with heterozygous germline mutations in CTLA4. Science. 2014;345(6204):1623-7.
8. Verloes A. Updated diagnostic criteria for CHARGE syndrome: a proposal. Am J Med Genet A. 2005;133A(3):306-8.
9. Coulter TI, Chandra A, Bacon CM, Babar J, Curtis J, Screaton N, et al. Clinical spectrum and features of activated phosphoinositide 3-kinase delta syndrome: A large patient cohort study. J Allergy Clin Immunol. 2017;139(2):597-606 e4.
10. Lucas CL, Kuehn HS, Zhao F, Niemela JE, Deenick EK, Palendira U, et al. Dominant-activating germline mutations in the gene encoding the PI(3)K catalytic subunit p110delta result in T cell senescence and human immunodeficiency. Nat Immunol. 2014;15(1):88-97.

Supplementary Figure. Filtering strategy of WES data and technical outcome of the WES data.

A. Filtering strategy. B. Total number of reads in each sample. C. Average read depth in each sample. D. Coverage of target regions in each sample (20X and 50X).

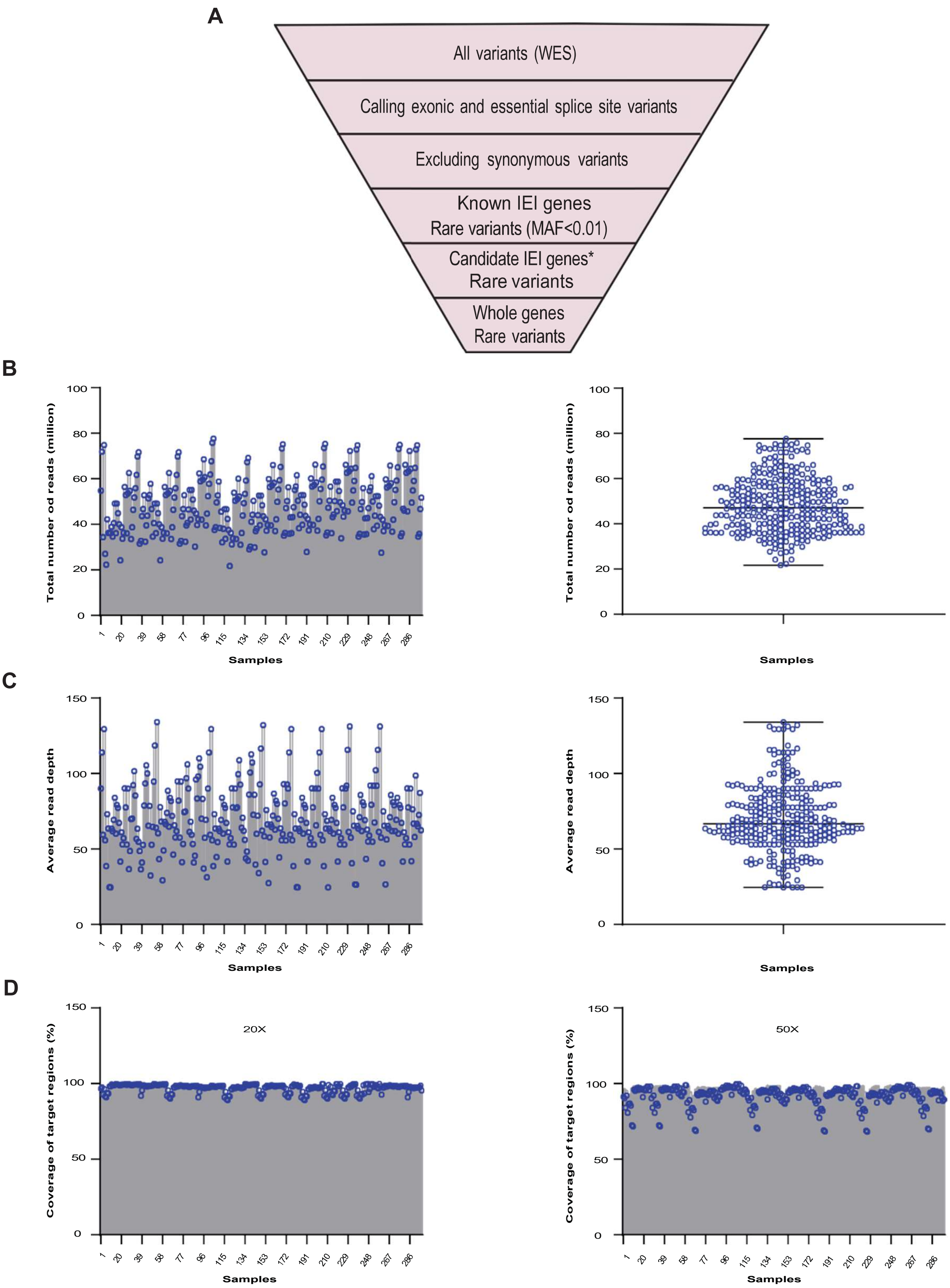
A
All variants (WES)
Calling exonic and essential splice site variants
Excluding synonymous variants
Known IEI genes
Rare variants (MAF<0.01)
Candidate IEI genes*
Rare variants
Whole genes
Rare variants
B
Total number od reads (million)
Samples
C
Average read depth
Samples
D
Coverage of target regions (%)
20X
50X
Samples
